# Metabolic and Environmental Conditions Determine Nuclear Genomic Instability in Budding Yeast Lacking Mitochondrial DNA

**DOI:** 10.1534/g3.113.010108

**Published:** 2013-12-27

**Authors:** Léon Dirick, Walid Bendris, Vincent Loubiere, Thierry Gostan, Elisabeth Gueydon, Etienne Schwob

**Affiliations:** * Institut de Génétique Moléculaire de Montpellier UMR 5535 CNRS, 1919 route de Mende, 34293 Montpellier cedex 5, France; † Université Montpellier 2, Place Eugène Bataillon, 34095 Montpellier cedex 5, France; ‡ Université Montpellier 1, 5 Bd Henri IV, 34967 Montpellier cedex 2, France

**Keywords:** mitochondrial DNA, nuclear genome instability, metabolism, membrane potential, calorie restriction

## Abstract

Mitochondrial dysfunctions are an internal cause of nuclear genome instability. Because mitochondria are key regulators of cellular metabolism, we have investigated a potential link between external growth conditions and nuclear chromosome instability in cells with mitochondrial defects. Using *Saccharomyces cerevisiae*, we found that cells lacking mitochondrial DNA (*rho0* cells) have a unique feature, with nuclear chromosome instability that occurs in nondividing cells and strongly fluctuates depending on the cellular environment. Calorie restriction, lower growth temperatures, growth at alkaline pH, antioxidants (NAC, Tiron), or presence of nearby wild-type cells all efficiently stabilize nuclear genomes of *rho0* cells, whereas high glucose and ethanol boost instability. In contrast, other respiratory mutants that still possess mitochondrial DNA (*RHO^+^*) keep fairly constant instability rates under the same growth conditions, like wild-type or other *RHO^+^* controls. Our data identify mitochondrial defects as an important driver of nuclear genome instability influenced by environmental factors.

Nuclear genome instability is a threat to normal cellular function, for example, in cancer or aging (Hanahan and Weinberg 2011; Maslov and Vijg 2009). Genomic instability can stem from either internal defects (*i.e.*, cell-cycle errors) or external factors, when genotoxic compounds are present in the environment of the cell (oxidants, chemicals, high-energy rays, and others) (Hanawalt 1998). Among internal sources of instability, dysfunctions of mitochondria also lead to nuclear genome instability (Karthikeyan and Resnick 2005; Veatch *et al.* 2009).

Nuclear genome instability in cells with mitochondrial dysfunction includes a modest increase of point mutations in nuclear genes, when the mitochondrial respiratory chain is impaired (Flury *et al.* 1976; Rasmussen *et al.* 2003). In a more recent study, a severe nuclear chromosome instability (CIN) phenotype was found in budding yeast cells completely devoid of mitochondrial DNA (Veatch *et al.* 2009). Mutations in genes encoding components of the respiratory chain, but still in possession of their mitochondrial DNA (*RHO^+^*), only led to a mild increase in nuclear CIN. Additional important conclusions were drawn from this study. First, in *rho0* cells, nuclear DNA damage stems from DNA breaks and mitotic recombination (rather than single nucleotide substitutions). Second, the decrease in mitochondrial membrane potential (and not the mere loss of respiratory capacity) is the main parameter influencing nuclear CIN in *rho0* cells. Third, the loss of mtDNA is a common event after replicative aging. Finally, a defect in iron–sulfur cluster (ISC) metabolism, which originates in mitochondria and is partially exported to the cytoplasm (Lill and Muhlenhoff 2008), might ultimately lead to nuclear DNA instability in these cells. Veatch *et al.* suggested that decreased levels of cytoplasmic ISCs, because of low mitochondrial membrane potential in *rho0* cells, impair the proper folding and maturation of a number of nuclear proteins required to maintain genomic stability (such as DNA Pol d and Rad3). This could provide a link between dysfunction in an organelle, the mitochondrion, and its consequences in another compartment, the nucleus, via an ISC-dependent pathway (Veatch *et al.* 2009). A cytoplasmic ISC cofactor, Mms19, was recently characterized and implicated in the maturation of enzymes involved in nuclear genome maintenance (Gari *et al.* 2012; Stehling *et al.* 2012; van Wietmarschen *et al.* 2012), providing a possible link between ISC defect and nuclear genome instability.

Mitochondria contain multiple copies of a DNA molecule (mitochondrial or mtDNA) carrying genes coding for components of the respiratory chain (complex III and IV), for the mitochondrial translation machinery, as well as for components of the FO subunit of mitochondrial ATP synthase. Other proteins required for mitochondrial function (estimated between 500 and 1000) are encoded by the nuclear genome and are imported in mitochondria (Contamine and Picard 2000; Sickmann *et al.* 2003). Alterations of mtDNA can span from single nucleotide mutations (*mit^−^*) to partial deletions (*rho^−^*) or full loss of all mtDNA molecules (*rho0*). Several conditions can promote the loss of mtDNA. Those include replicative aging (Veatch *et al.* 2009), nuclear mutations (*e.g.*, yeast Frataxin homolog) (Wilson and Roof 1997), defects in mitochondrial fusion process (Rapaport *et al.* 1998), and the presence of chemicals, including some antitumor drugs (Singh *et al.* 1992). Ethanol can also lead to mtDNA loss, as shown in mouse liver, as well as in heart, brain, and muscle cells (Demeilliers *et al.* 2002; Mansouri *et al.* 2001) and in wine-making flor yeast (Ibeas and Jimenez 1997). Cells lacking mtDNA maintain "proto-mitochondria," with abnormal shape (Holmuhamedov *et al.* 2003) and limited metabolism, but are still essential for cell survival. Maintenance of a mitochondrial membrane potential (ΔΨ) is essential, because it is involved in protein import into mitochondria and required for mitochondrial biogenesis (Baker and Schatz 1991). In *rho0* cells or in cells under anaerobic conditions, glycolytic ATP is imported into mitochondria and hydrolysis of ATP by the F1-ATPase activity is essential to generate a mitochondrial membrane potential and to maintain viability (Giraud and Velours 1997; Lefebvre-Legendre *et al.* 2003). This low and atypical mitochondrial membrane potential distinguishes *rho0* cells from other respiratory mutants with an intact ATP synthase.

Because mitochondria are central organelles for regulation of cellular metabolism, we asked whether cells with dysfunctional mitochondria could still deal with metabolic and environmental variations and, in particular, their capacity to maintain nuclear genome stability when growth conditions are modified. Here, we show that cells lacking mitochondrial DNA (*rho0*), but not other types of respiration-defective cells, display highly conditional nuclear genome instability, ranging from low/wild-type levels to very high instability, depending on growth parameters and cellular environment. Implications of this finding, comparisons with the current model, and additional hypotheses are presented.

## Materials and Methods

### Strain construction

**Figure 1:**
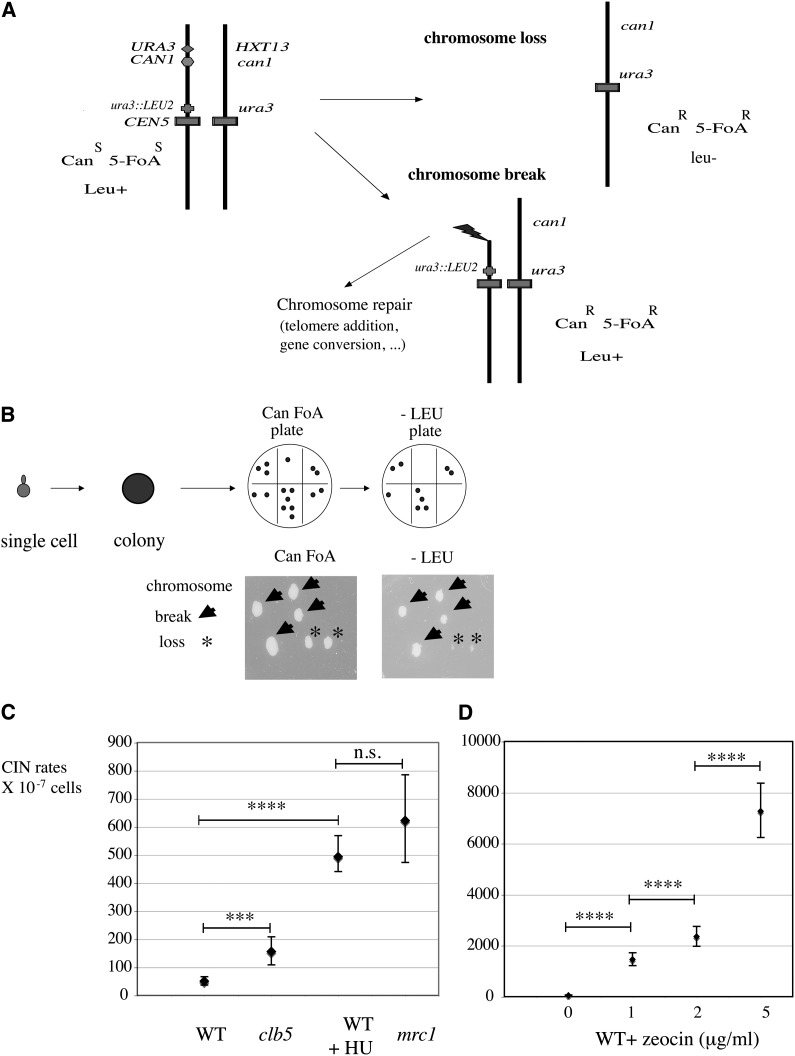
CINA, a sensitive and clonal assay to monitor chromosomal instability in yeast. (A) Genome instability is measured by positive selection of marker loss (*URA3* and *CAN1* genes) on the distal arm of chromosome V in diploid strains heterozygous for *CAN1* and *URA3*. Combined loss of *URA3* and *CAN1* genes leads to colonies resistant to the drugs 5-FoA and canavanine (Can FoA R). In CINA, this can be attributable either to chromosome break or to full chromosome loss. (B) Starting from single cells, clones are grown on solid medium and chromosome instability events detected on CanFoA plates. Replica on −LEU allows distinguishing between chromosome break (CanFoA-resistant, LEU^+^) and chromosome loss (CanFoA-resistant, leu^−^) . (C) CIN rates in untreated wild-type (WT) cells, in S-phase cyclin mutant *clb5*Δ, in WT cells grown in the presence of 50 mM hydroxyurea (+HU) and in S-phase checkpoint mutant *mrc1*Δ. (D) CIN rates in WT cells grown to colonies in the presence of zeocin. Note the differences in Y scale. All strains were grown on YEPD plates at 30°. Details of the assay and statistical analysis are described in *Materials and Methods*.

For CIN assay (CINA), a *LEU2* wild-type gene was integrated at the *ura3* locus (*ura3*::*LEU2*, pUL9) (Cross 1997) close to *CEN5* in an S288C strain carrying a *URA3-CAN1* selection cassette on the distal arm of chromosome V (RDKY3615) (Chen and Kolodner 1999). Integration of the marker was verified by southern blot and genetic analyses. The resulting strain was L1520: *MATa*, *ura3-52*::*LEU2*, *leu2Δ1*, *trp1Δ63*, *his3Δ200*, *lys2ΔBgl*, *hom3-10*, *ade2Δ1*, *ade8*, *hxt13*::*URA3-CAN1* . The mating partner carrying a *can1* mutant allele marked with kanMX4 is L1571: *MATalpha his3Δ1 leu2Δ0 met15Δ0 ura3Δ0 can1*::*kanMX4* (Euroscarf, BY4741). Mating of L1520 with L1571 and pulling of a diploid zygote led to L1577, the wild-type CINA strain (for schematic view, see Figure 1A). To generate a CINA strain with a given nuclear mutation, the mutation of choice was introduced separately in two haploid CINA parents by genetic crosses. One CINA parent is L1847, a *MATalpha* version of L1520. The nuclear mutant was also crossed to the second CINA parent carrying an untagged *can1* mutation (L1607: *MATalpha leu2Δ0 lys2Δ0 met15Δ0 trp1*::*63 ura3Δ0 can1*). The correct segregants are crossed and lead to a diploid CINA strain with the homozygous deletion for the nuclear gene and appropriate heterozygous selection markers (*CAN1/can1URA3*::*hxt13/HXT13ura3*::*LEU2/ura3*) for chromosome instability assay. All CINA strains used in this study are in the S288C strain background and the mutants marked with *KanMX4* are from the Euroscarf collection of deletion strains (Supporting Information, Table S1).

To generate *rho0* strains, cells were grown to saturation in liquid YPD medium plus ethidium bromide (10 μg/ml) and plated on YPD plates for individual colonies. Clones were checked for growth defects on a nonfermentable carbon source (YPG 2% glycerol) and absence of mtDNA was confirmed by DAPI staining. To further confirm the absence of mtDNA, two distant mitochondrial DNA loci, *COX3* and *OLI1*, were checked by PCR (COX3dir:GGT AAT ATG AAT ATG GTA TAT TTA GC; COX3rev: GTT ACA GTA GCA CCA GAA GAT AAT AAG; OLI1dir:ATG CAA TTA GTA TTA GCA GCT AAA T; OLI1rev: CCG AAT AAT AAT AAG AAT GAA ACC ATT A). *RAD5* locus was used as a positive control for a nuclear gene. For assays on newly born *rho0* cells, exponentially growing *RHO^+^* cells were treated with ethidium bromide (30 μg/ml) for 6 hr in YEPD, washed, resuspended in fresh medium for 15 hr, and single unbudded cells were micro-manipulated on plates for colony growth. To obtain spontaneous *rho0* mutants, untreated wild-type cells were plated to single colonies on YEP plates containing 4% glycerol and 0.1% glucose. This enhances the growth differences between *RHO^+^* and *rho^−/0^* cells. Very slow-growing clones were detected under the microscope, recovered by micromanipulation, and further checked by DAPI stain and PCR reaction for absence of mitochondrial DNA. The *RHO^+^* status of respiratory chain mutants (*e.g.*, *cyt1*) was checked by complementation with a *rho0* strain. Unless stated, all strains were grown at 30° on YEPD plates (2% glucose).

### CINA

For clonal assays, single cells from CINA strains were micromanipulated on solid medium at regular intervals of 0.5 cm, giving rise to nonsolitary colonies (approximately 50 colonies/plate). When grown to a visible size (approximately 10^7^ cells), a representative colony was picked and measured both for cell number and cell size (CASY-1 TTC Cell Analyzer; Schärfe, Germany), and a dilution was plated on YEPD to measure viability. Individual colonies were diluted to allow plating of the chosen number of cells (1–5 × 10^5^ cells, depending on rate of nuclear instability) and allowed to grow for 3 d at 30° on selective SD minus arginine plates containing canavanine 60 mg/L (L-canavanine sulfate; Sigma) and 5-fluororotic-acid 0.5 g/L (Toronto Research Chemicals) to select for cells with rearranged genomes. CanFoA plates: Yeast minimal SD-Arg medium/L: 2 g yeast nitrogen base without amino acids; 5 g ammonium sulfate; 20 g agar; 50 mg tyrosine; 30 mg isoleucine; 30 mg methionine; 50 mg phenylalanine; 100 mg tryptophan; 25 mg histidine; 50 mg leucine; 50 mg lysine; 50 mg tryptophan, 25 mg uracil, 25 mg adenine. Plating adjustment of the cell numbers were performed depending on the genomic instability of individual strains. Using this protocol, six assays can easily be performed on a single (10-cm) Petri dish. Rates of instability were calculated by fluctuation analysis of the median by the maximum likelihood method and represented as the median with 95% confidence interval using FALCOR online webtool (Hall *et al.* 2009). Rates are expressed per 10^7^ viable cells. Data sets were also analyzed using Lea-Coulson estimator and *P* values were calculated using the Mann-Whitney nonparametric test. Statistical significances are indicated in graphs by the following: ns, not significant, *P* > 0.05; *P ≤ 0.05; **P ≤ 0.01; ***P ≤ 0.001; and ****P ≤ 0.0001. At least 24 independent clones were analyzed for each point. Chromosome breakage can be distinguished from full chromosome loss by replica plating of CanFoA-resistant clones on minus leucine plates (Figure 1B) (−LEU). CanFoA-resistant Leu^+^ clones were the result of chromosomal breaks between the *URA3-CAN1* and *LEU2* markers, whereas CanFoA-r leu^−^ clones resulted from full chromosome loss of the homolog carrying the selective markers (Figure 1). Low-frequency meiosis could lead to false-positive results for CIN (CanFoA-resistant). Unlike the genuine diploid clones, these are haploids and can be measured using mating type tests. Maters can stem from meiotic recombination or from the loss of chromosome III, the chromosome carrying the mating type locus. For a wild-type strain, positive maters ranged from 1% to 5% of total CIN clones, making them negligible in the global percentage of chromosome instability values. The *rho0* cells do not sporulate; therefore, they did not suffer from this small bias.

## Results

### CINA, a sensitive assay to quantify chromosome instability in yeast

To test nuclear genome instability in a large number of samples, we designed a quantitative and highly sensitive assay called CINA (Figure 1). It is based on the gross chromosomal rearrangement (GCR) assay (Schmidt *et al.* 2006) but is 10,000-times more sensitive. To improve the sensitivity, we designed the assay based on diploid cells (Figure 1A) with the following features: as in the original GCR assay, chromosome instability is measured by positive selection of two marker losses (*URA3* and *CAN1*, linked in the distal part of chromosome V) (Chen and Kolodner 1999); it uses a diploid, allowing either breakage or full chromosome loss to occur without loss of viability, in contrast to the original GCR assay in which haploid cells can only break within a limited area around the *URA3-CAN1* markers and survive; the addition of a *LEU2* marker close to CEN V allows the ability to distinguish between chromosome breakage and chromosome loss (Figure 1B) and CINA is performed starting from single cells grown to colonies on the plate of choice (clonal assay). Micromanipulation of single cells allows for homogenous distance/settings between all clones. Because it starts from single cells, CINA measures *de novo* instability events rather than accidental amplification of preexisting rearrangements present at t = 0 in assays in which large inoculums are required.

When grown on YEPD plates, wild-type cells display CIN rates of approximately 50 marker losses per 10 million cells (51 × 10^−7^ CanFoA-resistant/cell) (Figure 1C), *i.e.*, approximately 10,000-fold higher than in the GCR assay; approximately two-thirds of events are attributable to chromosome breaks (Can^R^FoA^R^/68% LEU^+^) and one-third are attributable to chromosome loss (Can^R^FoA^R^/32% leu) (Figure S1). To validate the assay, we used drugs and mutations that affect chromosome stability in yeast. Genomic destabilization can be detected in the cell-cycle mutant *clb5* (S-phase cyclin), increasing three-fold (156 × 10^−7^; *P* < 0.0001) (Figure 1C). Hydroxyurea (HU), an inhibitor of ribonucleotide reductase Rnr1p, slows replication forks (Alvino *et al.* 2007) and increases chromosome instability 10-fold in the CINA (493 × 10^−7^ at 50 mM HU), similar to the rate observed in the S-phase checkpoint mutant *mrc1* (622 × 10^−7^) (Figure 1C). Wild-type colonies grown in the presence of zeocin, a drug that creates double-strand breaks, strongly increased CIN in a dose-dependent manner, 28-fold (1461 × 10^−7^) at 1 μg/ml and 140-fold at 5 μg/ml (7270 × 10^−7^) (Figure 1D). Thus, CINA is a sensitive assay that allows the quantitative detection of chromosome instability in small samples, including in small size colonies/clones (see *Materials and Methods*).

### *Rho0* cells are genetically unstable when grown under standard rich growth conditions

**Figure 2:**
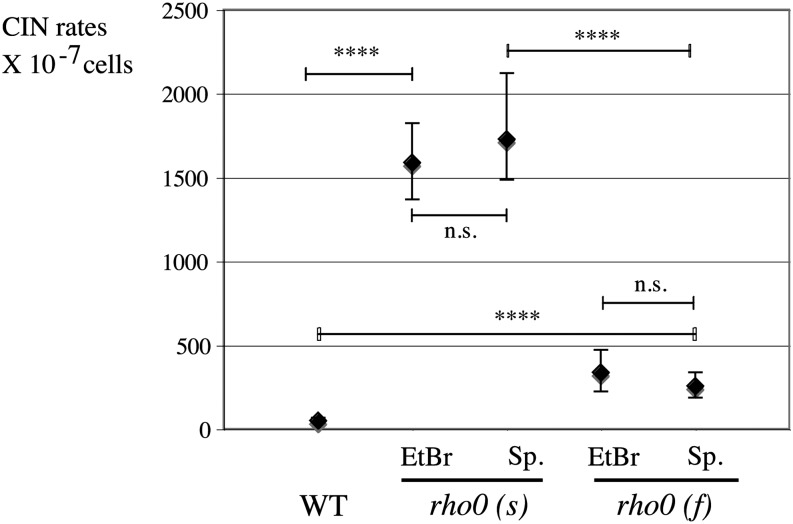
Established *rho0* strains display elevated nuclear chromosome instability in CINA. Strains lacking mitochondrial DNA were obtained by either ethidium bromide treatment (EtBr) or in absence of any treatment (spontaneous, *rho0* sp.; see *Materials and Methods*) and tested many divisions after the original mtDNA loss event. Two subtypes of *rho0* clones, slow-growing and fast-growing *rho0* (Figure S1) were analyzed separately [*rho0* (s) and *rho0* (f)]. CIN rates in wild-type (L1577), in slow-growing *rho0* (s) [EtBr (L1993) and in spontaneous (sp., L2232)], and in fast-growing *rho0* (f) clones [(EtBr (L1994) and spontaneous (sp., L2249)].

To precisely quantify the rates of nuclear genome instability in cells lacking mitochondrial DNA (*rho0* cells), we isolated *rho0* clones starting from the diploid CINA parental strain (L1577; S288c background) (Table S1); nuclear chromosome instability was then analyzed many generations after the mtDNA loss event (“established” *rho0* clones). *Rho0* cells were first obtained after ethidium bromide treatment (Slonimski *et al.* 1968). When plated, these cells (all *rho0*) showed a dimorphic colony growth phenotype, with slow-growing and fast-growing clones (L1690) (Figure S2). A similar observation was made with newborn *rho0* cells, also in the S288c genetic background (Veatch *et al.* 2009). Subcloning of slow clones gave rise to a mixture of slow-growing and fast-growing colonies [*rho0* (s), L1993], whereas subcloning of fast clones only yielded fast-growing ones [*rho0* (f), L1994] (Figure S2A). The frequency (approximately 1 in 10 cells) of conversion from slow-growing to fast-growing clones suggests an efficient suppression of the slow colony growth phenotype after mtDNA loss. No reverse switching from fast-growing to slow-growing clones was observed. Because of this obvious dimorphic growth phenotype, we measured chromosome instability in slow and fast *rho0* strains. By CINA, slow-growing *rho0* (s) have a very high rate of nuclear chromosome instability, approximately 30-fold more than the *RHO+* (wild-type) level (Figure 2) [*rho0* (s) EtBr = 1590×10^−7^] or three-fold more than that in *mrc1* mutants (Figure 1C), when tested under standard conditions (YPD medium, 30°). A faster growing subclone (L1994) was isolated from the same strain (L1993); it showed a lower (approximately six-fold) but still highly significant increase in CIN compared to wild-type [*rho0* (f) EtBr = 338 × 10^−7^; *P* < 0.0001] (Figure 2). To confirm this result using an unbiased method to recover *rho0* cells, we searched for spontaneous *rho0* clones (*rho0* sp.) in untreated wild-type populations (see *Materials and Methods*). The *rho0* strains recovered in this way behaved just like ethidium bromide–induced *rho0* cells. Both slow-growing and fast-growing clones were found, with the same unidirectional switch from slow-growing to fast-growing clones (Figure S2B). CIN assay on slow-growing (L2232; *rho0* slow, sp.) and fast-growing (L2249; *rho0* fast, sp.) clones gave the very same CIN pattern as measured for ethidium bromide–induced clones (Figure 2) [*rho0* (s), sp.= 1729 × 10^−7^; *rho0* (f), sp.= 257 × 10^−7^; *P* < 0.0001]. No statistical difference was measured between *rho0* strains obtained by ethidium bromide treatment compared to spontaneous *rho0* (*P* = not significant) (Figure 2). To distinguish between chromosome breaks and chromosome loss, CanFoA-resistant colonies were patched and checked for the presence of the *LEU2* marker; in *rho0* cells, the majority of CIN stemmed from a chromosome break rather than a loss [more than 80% breaks both in *rho0 (s)* and *rho0 (f)*] (Figure S3, A and B). Contrary to CIN, point mutation rates in haploid *rho0 (f)* cells in our strain background remained unchanged compared to wild-type (Figure S4). In summary, established *rho0* cells display a high rate of nuclear genome instability (mostly chromosome breaks) with two modes: very high CIN in slow-growing *rho0* and a more moderate yet highly significant increase in faster growing *rho0* cells. Thus, nuclear chromosome breaks occur at high rates when *rho0* cells are grown to colonies under optimum, rich growth conditions.

### Nuclear DNA damage in *rho0* cells occurs mostly in nondividing cells

**Figure 3:**
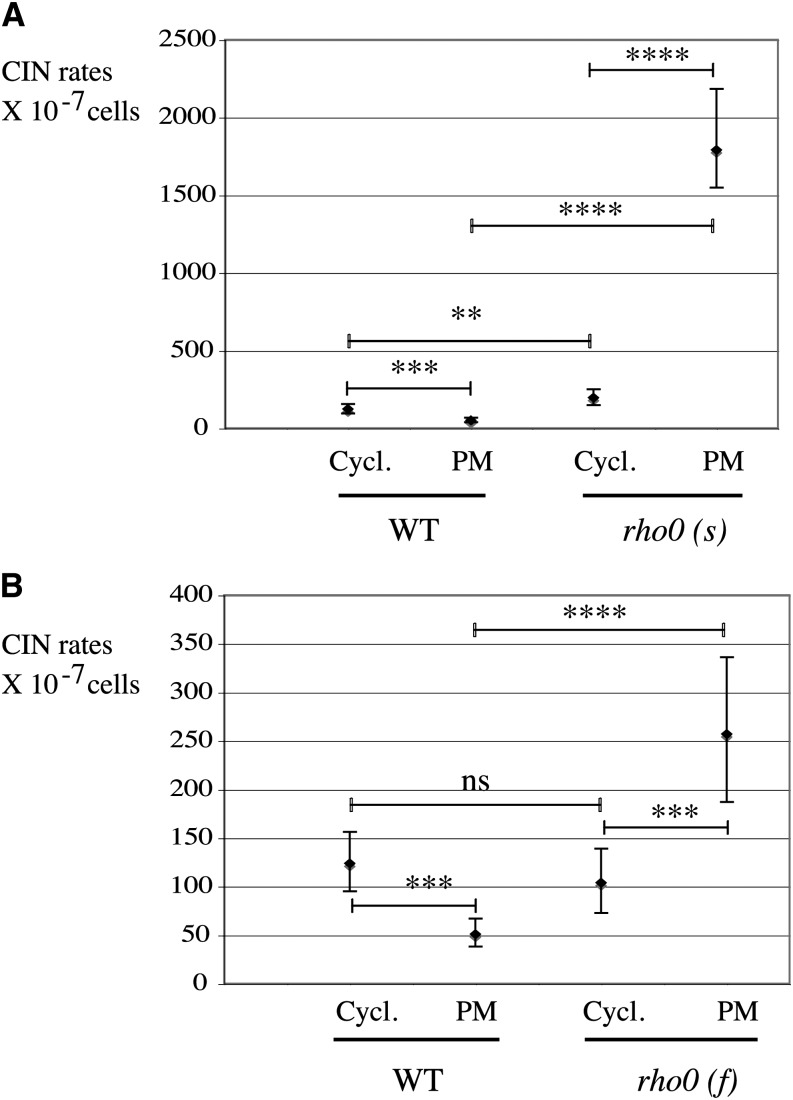
High chromosome instability in *rho0* cells does not occur during exponential growth phase but in postmitotic (PM) cells. Wild-type (WT) and *rho0* cells [*rho0* (s) and *rho0* (f)] were grown on YPD plates at 30° for 42 hr (Cycl., cycling) or for 4 d (PM) and tested in CINA. (A) Comparison of WT and slow *rho0* (s) (L2232). (B) Comparison of WT and fast *rho0* (f) (L2249)

We first explored when in the life cycle of *rho0* cells that nuclear genome instability arises. Instability stemming from DNA replication/repair defects (internal sources) is expected to occur in dividing cells, whereas the presence of genotoxic compounds in the environment (external sources) can trigger instability in both dividing and nondividing cells. We thus compared CIN rates in cycling compared with nondividing [postmitotic (PM) cells]. When grown on solid media, nonsolitary yeast clones (intercolony distance <3 cm) behave like exponentially growing cells for the first 42 hr (Meunier and Choder 1999). Log phase (42 hr after plating on YPD) and PM cells (4-day-old clones) therefore were compared for CIN rates, both in wild-type and in *rho0* strains. Wild-type CIN rates decreased moderately but significantly (*P* = 0.0001) between cycling (124 × 10^−7^) and PM (51 × 10^−7^) conditions, as expected (Figure 3, A and B). On the contrary, genome instability in *rho0* strains remained surprisingly low in cycling cells and was increased in 4-day-old PM colonies. The effect was very striking in slow-growing *rho0* cells [*rho0 (s)* cycling = 194 × 10^−7^ *vs.* PM = 1792 × 10^−7^] (Figure 3A), but was also significant in fast-growing *rho0* cells [*rho0 (f)* cycling = 104 × 10^−7^; PM = 257 × 10^−7^; *P* = 0.0003] (Figure 3B). Importantly, in cycling populations, CIN in *rho0* cells did not differ from wild-type [not at all for *rho0 (f)*, less than two-fold for *rho0 (s)*] (Figure 3, A and B).

Thus, nuclear instability in *rho0* cells occurs only in nondividing cells, after an exponential phase on rich medium, as if genotoxic compound(s) had appeared in the cell environment. Because the environment was not experimentally modified, these “compounds”/genotoxic effects are likely the result of the colony’s metabolic activity and/or of the modification of the physiology of the cell. Therefore, we next analyzed the effect of changing environments/metabolic conditions on nuclear CIN in *rho0* cells in comparison to *RHO+* strains.

### Environmental conditions can efficiently stabilize the nuclear genomes in *rho0* cells: effects of low temperature and calorie restriction

The high nuclear genome instability described occurred in standard growth conditions, *i.e.*, in rich medium with high glucose (2%) and optimum temperature (30°). Because mitochondria are key players of cell metabolism, we asked whether modifications of metabolic or environmental conditions could influence the capacity of mitochondria-defective cells to maintain nuclear genome stability. We chose parameters based on their known effects on cellular fitness or longevity (*e.g.*, calorie restriction) or on global metabolism (growth t°).

**Figure 4:**
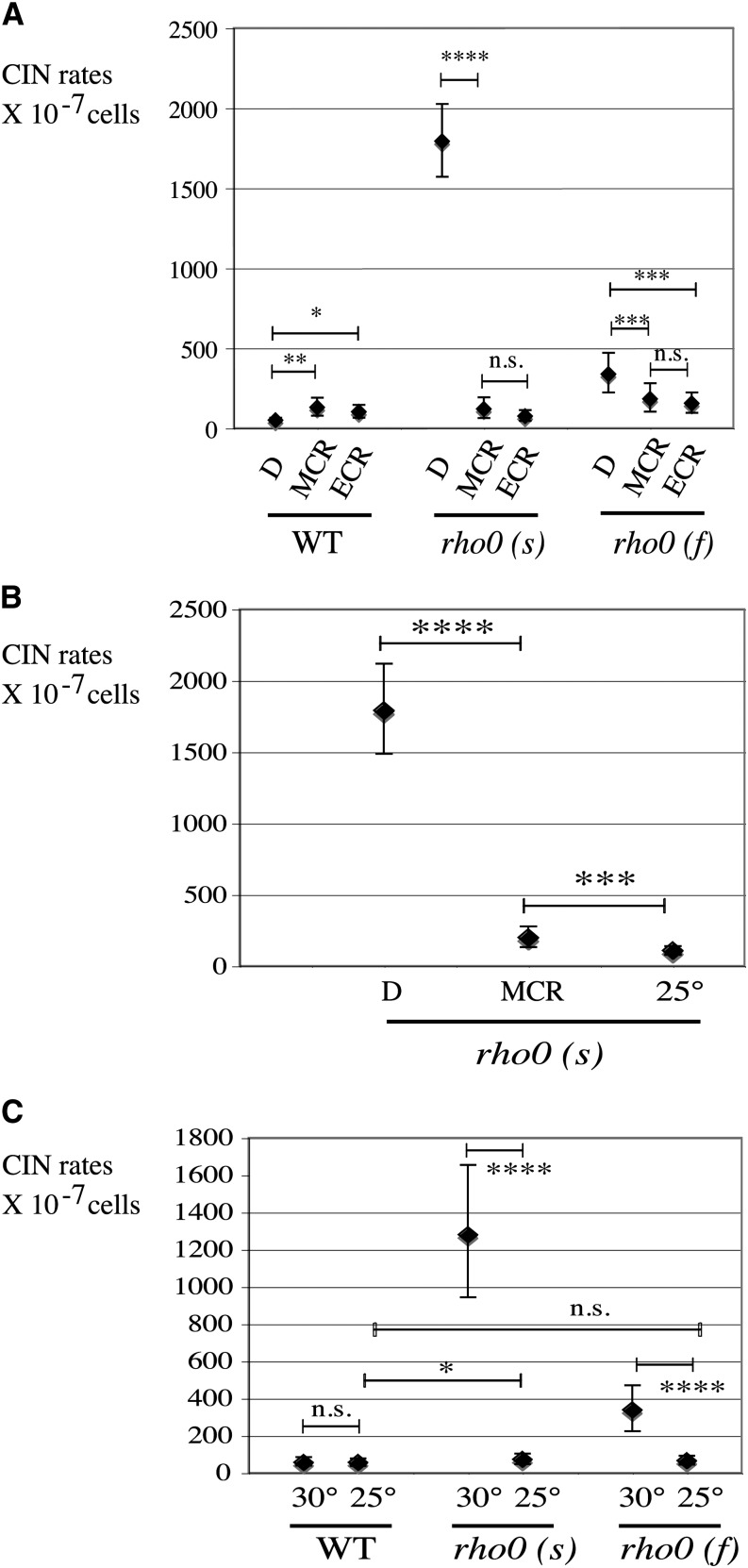
CIN-stabilizing factors. Calorie restriction or low growth temperatures strongly reduced nuclear CIN in *rho0* but not in wild-type cells. (A) CIN in *RHO^+^* strains (WT L1937), *rho0* (s) (L1993), and *rho0* (f) (L1994) under regular (2%) glucose YEPD, moderate calorie restriction (MCR; YEP + 0.5% glucose), and extreme calorie restriction (ECR; YEP + 0.05% glucose). (B) Stabilization of the nuclear genome in spontaneous slow *rho0* strain [*rho0* (s); L2232] by MCR or low growth temperature (25°). (C) Effect of growth temperature on CIN rates in WT (L1937) and spontaneous *rho0* [*rho0* (s), L2232; *rho0* (f), L2249] tested on YEPD (2%) at 30° and 25°.

Calorie restriction is known to increase life span and organismal fitness (Anderson and Weindruch 2010; Fontana 2009; Longo and Fontana 2010). Strikingly, we found that calorie restriction also has very beneficial effects on the stability of nuclear genomes in *rho0* cells. CIN reduction is already very strong in slow *rho0* cells grown under moderate calorie restriction (MCR; 0.5% glucose; CIN = 121 × 10^−7^/cells) or at lower glucose concentration (CIN = 76 × 10^−7^/cells in ECR; 0.05% glucose) compared to standard 2% glucose concentrations (CIN = 1792 × 10^−7^/cells) (Figure 4A). Nuclear genomes in spontaneous *rho0* cells were also stabilized efficiently by growth under MCR conditions (Figure 4B). However, unexpectedly, calorie restriction did not reduce CIN rates in the wild-type *RHO^+^* strain (Figure 4A). Instead, a mild CIN increase was measured in wild-type after growth under MCR (CIN = 129 × 10^−7^/cells; *P* = 0.004) and extreme (CIN = 102 × 10^−7^/cells; *P* = 0.040) compared to standard 2% glucose concentration (CIN = 51 × 10^−7^/cells) (Figure 4A).

Another global means of tuning metabolism is modifying the growth temperature. We therefore repeated CINA on *rho0* cells grown on YEPD (2% glucose) at 25°, which is the low edge for optimal growth temperature in *S. cerevisiae* (Watson 1987). Remarkably, this moderate temperature decline reduced nuclear genome instability with great efficiency, again in *rho0* cells only but not in wild-type cells (Figure 4C). Nuclear genomes were stabilized at 25° both in slow-growing (CIN 30° = 1281 × 10^−7^ *vs.* CIN 25° = 74 × 10^−7^) and fast-growing (CIN 30° = 338 × 10^−7^ *vs.* CIN 25° = 66 × 10^−7^) *rho0* cells to wild-type levels (CIN 30° = 57 × 10^−7^ *vs.* CIN 25° = 56 × 10^−7^). Again, *rho0* cells showed a highly conditional CIN phenotype in response to temperature, unlike wild-type cells. We conclude that reducing growth conditions from optimum parameters to either lower glucose concentrations or lower growth temperature strongly stabilizes the nuclear genome in cells lacking mitochondrial DNA, both in *rho0 (s)* and in *rho0 (f)*, but not in *RHO*^+^, cells.

### Factors with a negative impact on genome stability in *rho0* cells

**Figure 5:**
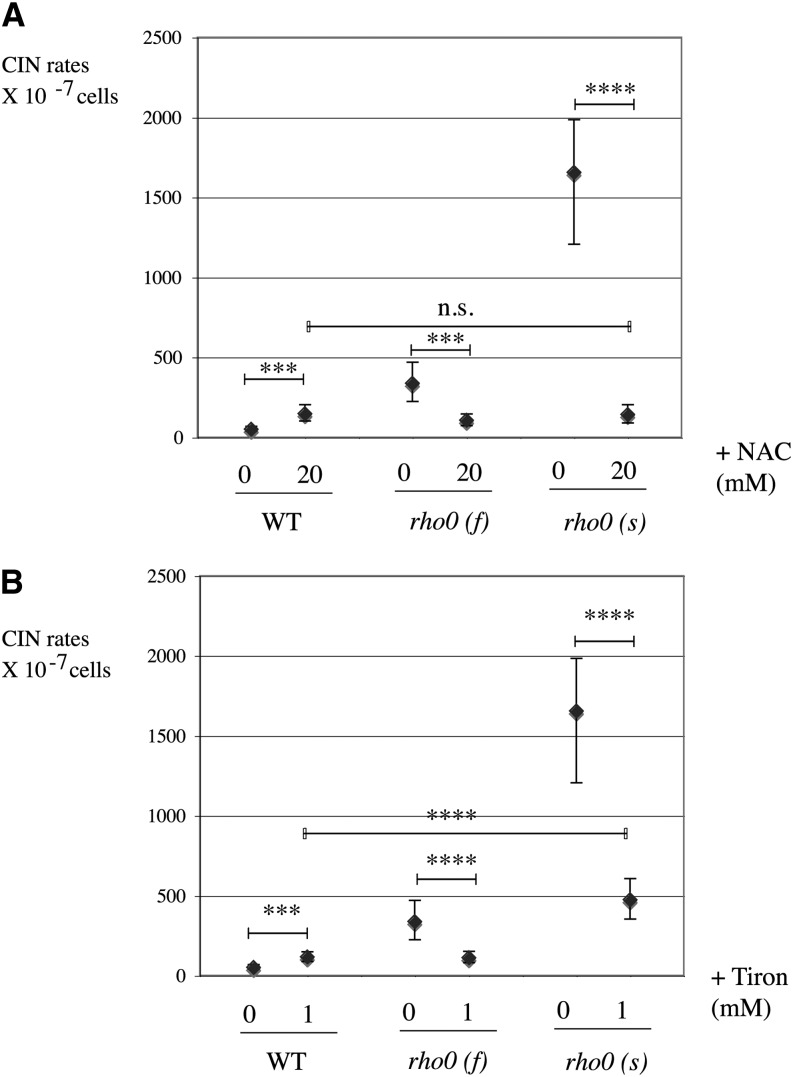
Nuclear genome stabilization in rho0 cells grown in the presence of antioxidants. (A) Wild-type (WT; L1577) and slow *rho0* cells (L1690) grown at 30° on YEPD medium in the presence of 20 mM of N-acetyl-cysteine (NAC) and tested in CIN assay. Addition of NAC strongly decreases genome instability of *rho0* cells, close to WT levels. (B) The same effect on CIN is obtained by addition of electron scavenger Tiron at 1 mM on fast *rho0* strain [*rho0* (f); L1994].

To search for conditions that, on the contrary, could negatively impact *rho0* cells, we looked for drugs that differentially affect cell survival between wild-type and *rho0* strains. Tested drugs included an inhibitor of DNA synthesis (HU), a DNA alkylating agent [methylmethanesulfonate (MMS)], a DNA double-strand break inducer (bleomycin), and a microtubule poison (benomyl). As control strains, wild-type (checkpoint proficient) and checkpoint mutants for DNA damage/DNA replication *mec1sml1* and spindle checkpoint mutant *bub1* were used. Both *rho0 (f)* (L1994) and *rho0 (s)* (*mip1*, L1779) were tested. Figure S5A shows that *rho0* cells are not hypersensitive to any of the tested drugs, compared to wild-type. This suggests that *rho0* cells have no major defects in sensing, signaling, or repairing these types of damage. Consistently, the Rad53-dependent checkpoint was found to be functional in yeast cells lacking mitochondrial DNA (Crider *et al.* 2012). In contrast, *rho0* cells show striking growth defects when plated on media containing hydrogen peroxide (Figure S5, A and B), as previously reported (Grant *et al.* 1997), unlike wild-type and *bub1* control strains that formed colonies in the same conditions. Interestingly, *RHO*^+^ *mec1sml1* were as sensitive as *rho0* cells to H_2_O_2_ (Figure S5A). Unlike peroxide, *rho0* cells were not hypersensitive to the superoxide-generating agent menadione (Figure S5C). Hence, *rho0* cells seem particularly sensitive to oxidative stress by peroxide but not to the other drugs tested. Because *rho0* cells are sensitive to oxidants, we wondered whether intracellular oxidative stress might be responsible for the elevated CIN observed in unperturbed *rho0* cells (Figure 2). If such were the case, then one might expect to reverse the effect by the mere addition of an antioxidant to the medium. Addition of the oxygen scavenger and redox modulator N-acetyl cysteine (NAC) to the medium strikingly lowered the high CIN of *rho0* cells, both in slow and fast *rho0*. Most strikingly, at 20 mM NAC, CIN in *rho0 (s)* became indistinguishable from CIN in wild-type (*P* = not significant) (Figure 5A). Addition to the medium of another antioxidant, the electron-scavenger Tiron (Taiwo 2008), also stabilized nuclear genomes in both fast and slow *rho0* strains (Figure 5B). Although the stabilizing effect of Tiron on slow *rho0* is very significant (*P* < 0.0001), it was not quite as efficient as with NAC, and it remained significantly higher in *rho0 (s)* compared to wild-type (Figure 5B). Again, wild-type cells behaved differently from *rho0* cells because addition of either NAC (20 mM) or Tiron (1 mM) slightly increased CIN in both instances (two-fold; *P* = 0.011 and 0.002, respectively). Thus, *rho0*, but not wild-type cells, undergo far less genomic instability in the presence of an antioxidant. These data suggest the presence of an unidentified oxidation-dependent damage in *rho0* cells, leading to high genomic instability in standard conditions.

To explore this phenomenon further, we deleted the thioredoxin peroxidase gene *TSA1*, encoding a major ROS detoxifying enzyme with an important function of protecting from nuclear genome instability (Chae *et al.* 1994; Iraqui *et al.* 2009; Ragu *et al.* 2007). Either wild-type or *rho0* cells lacking *TSA1* were challenged with sublethal H_2_O_2_ (1 mM), a concentration that mildly increased CIN (two-fold) in wild-type and left CIN in *rho0 (f)* cells unchanged (Figure S6A). Control *tsa1* cells showed high CIN (25-fold more than wild-type), as described (Ragu *et al.* 2007), also moderately increased by H_2_O_2_ (two-fold) (Figure S6A). Removing *TSA1* from a *rho0 (f)* had a very strong impact on nuclear CIN. First, the basal level of CIN in *tsa1 rho0* was much higher [80-fold more than wild-type CIN levels, 16-fold more than *rho0 (f)*] (Figure S6A). Second, the mild H_2_O_2_ treatment further increased this instability to extreme proportions (400-fold more than wild-type) (Figure S6A). Despite their sensitivity at high peroxide concentrations (4 to 6 mM), we observed that *rho0* cells still possessed efficient CIN protection against peroxides at sublethal doses [1 mM; *rho0 (f)*± H_2_O_2_]; however, when their defense against peroxides was crippled (by deletion of *TSA1*), *rho0* cells became extremely prone to genome instability [*tsa1 rho0 (f)* ± H_2_O_2_] (Figure S6A).

**Figure 6:**
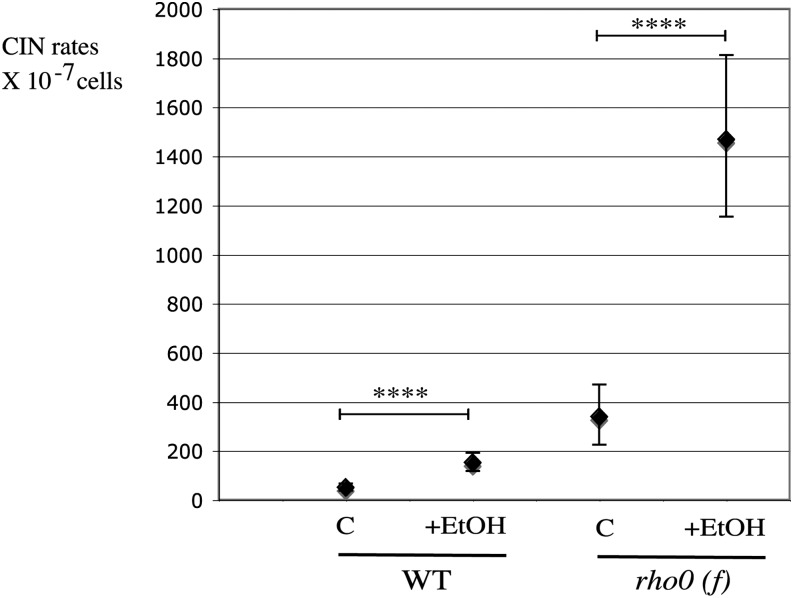
Genotoxic effect of ethanol on wild-type and *rho0* cells. Wild-type (L1937) or fast *rho0* (L1994) cells were grown on YPED (2% glucose) at 30° either in the absence (C) or in the presence of ethanol 2% (+EtOH).

In addition to its sensitivity to hydrogen peroxide, we found that *rho0* cells are also hypersensitive to the presence of ethanol. Ethanol, the main fermentation product in yeast, cannot be processed in the absence of a functional respiratory chain; therefore, *rho0* cells do not grow with ethanol as a sole carbon source. Both slow-growing and fast-growing *rho0* cells showed a marked sensitivity when a mild concentration of ethanol (2%) was added to YEPD (2% glucose) medium (YEPD plus ethanol) (Figure S7A). Growth of *rho0 (f)* was slowed but could be used for CINA (Figure S7B). Those *rho0* colonies grown on glucose plus ethanol medium were not suppressors, because they behaved just like the parental strain after a second passage on ethanol-containing medium (Figure S7B, passage 2). In *rho0 (f)*, ethanol further increased the nuclear genome instability five-fold, from 338 × 10^−7^ to 1468 × 10^−7^ (Figure 6), *i.e.*, up to the high level observed in highly unstable *rho0 (s)* strain. Moreover, the ratio between chromosome breaks and loss was not grossly affected by exposure to ethanol (Figure S7C). At the same concentration, ethanol had no gross effect on wild-type growth (Figure S7A), and a moderate but significant (two-fold to three-fold) increase in CIN rate was observed in the same conditions (Figure 6). In summary, exogenous factors with a negative impact on nuclear genome of *rho0* cells include ethanol as well as hydrogen peroxide (but only in the absence of the protection by thioredoxin enzymes). Finally, the presence of antioxidants can efficiently stabilize the nuclear genome of *rho0* cells, showing the importance of an unidentified oxidant-dependent reaction in the process of nuclear DNA damage in *rho0* cells.

### The peroxiredoxin mutant *tsa1* behaves differently than *rho0* cells

Another potential explanation for the beneficial effect CIN-stabilizing factors (MCR, low t°, and others) on *rho0* cells could be the reduction of an oxidative-dependent damage. CIN in *rho0* is very much reduced by antioxidants (Figure 5). To assess the potential *in vivo* benefits of slower metabolism in cells with a high oxidative load, we used a mutant in one of the main H_2_O_2_ detoxification enzymes, *TSA1*, known to be prone to high CIN (Ragu *et al.* 2007). We might expect to reduce CIN in *tsa1* mutants by slowing metabolism, just like in *rho0* cells. Under standard growth conditions, CIN in the *tsa1* mutant is 30-fold higher [in the same range as in *rho0 (s)*]; however, unlike in *rho0* cells, growth under MCR or at 25° did not stabilize CIN in *tsa1 RHO*^+^ cells (Figure S6B). Thus, these environmental conditions do not simply counteract a peroxide-dependent nuclear DNA damage. The addition of previously tested compounds, including NAC, ethanol, or peroxide, all led to a moderate CIN increase (less than two-fold). However, extreme calorie restriction (ECR) or growth on a poor carbon source, such as glycerol, reduced CIN quite efficiently, from 1424 × 10^−7^ (D) to 391 × 10^−7^ (ECR) and 142 × 10^−7^ (glycerol). Globally, CIN in *tsa1* appears to be fairly invariant, like in other *RHO*^+^ strains, with the exception of ECR, which brings significant benefit to the stability of that strain. Thus, MCR or low growth temperature is not sufficient to reduce DNA damage in *RHO*^+^ cells with a high oxidative load.

### Presence of a wild-type cell neighbors stabilizes *rho0* clones

The *rho0* cells are not born in an isolated environment but are surrounded by *RHO*^+^ cells. We therefore wished to investigate the effect of wild-type *RHO*^+^ cells on nuclear CIN in nearby *rho0* cells. Because of multiple and opposite influences of the environment on genomic CIN in *rho0* cells, as described, one cannot easily predict the outcome of such an experiment (for instance, glucose exhaustion would create a calorie restriction zone and reduce CIN, but production of ethanol would increase CIN).

**Figure 7:**
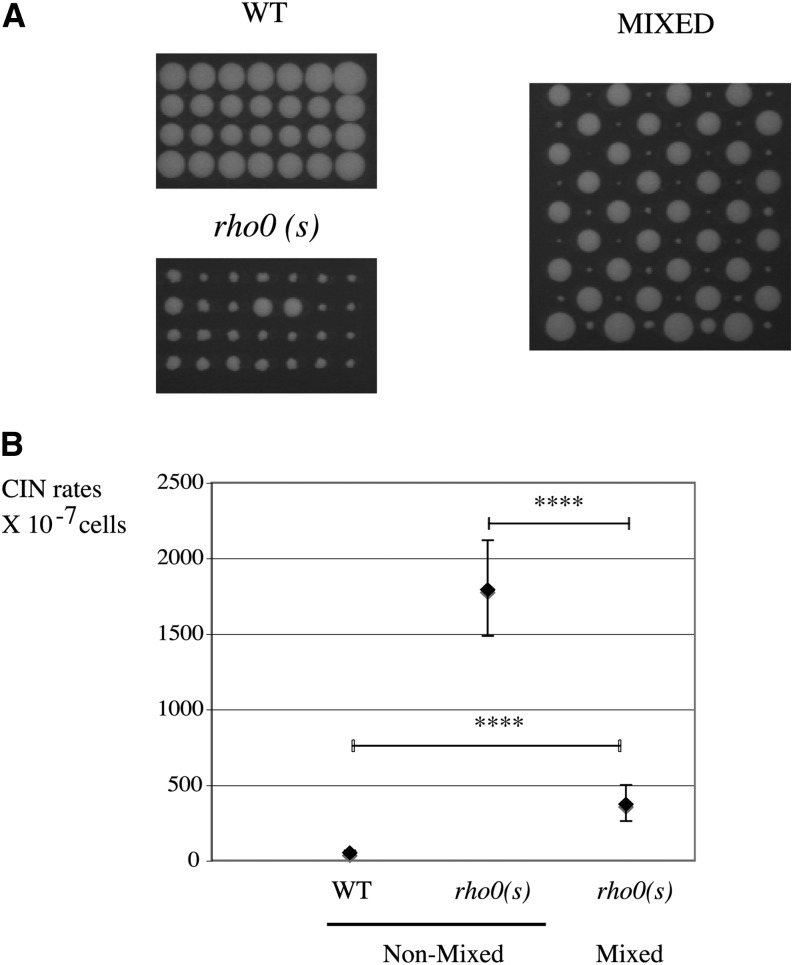
Nuclear genomes in *rho0* (s) clones are stabilized by the nearby presence of wild-type (WT) colonies. (A) Single cells from WT (L1937) and *rho0* (s) (L2232) were grown to colonies on separate plates [WT and *rho0* (s)] or micromanipulated on the same plate at alternate positions (mixed). (B) Slow-growing clones from mixed plate [*rho0* (s), mixed; L2232] were assayed for CIN and compared to the same strain grown alone [*rho0* (s), nonmixed; L2232].

At an intercolony distance less than 3 cm (0.5 cm in our experimental settings), colonies are of non-solitary type and can therefore influence each other (Meunier and Choder 1999). To assess whether CIN in *rho0* could be influenced by the proximity of wild-type cells, we alternatively micro-manipulated cells of wild-type and *rho0 (s)* genotypes (Figure 7A, mixed) on rich medium at 30° (high instability conditions for a *rho0* cell) and compared them to plates containing unmixed clones (Figure 7A) [wild-type and *rho0 (s)*]. Genomes of highly unstable *rho0 (s)* cells are clearly stabilized when surrounded by wild-type clones [*rho0 (s)*, mixed], still more than wild-type levels but close to the CIN level typical of the more stable *rho0 (f)* (nonmixed) (Figure 7B). We conclude that global modifications to the environment by wild-type cells lead to a clear decrease in nuclear DNA damage in nearby *rho0* clones.

### The nuclear genome in newborn *rho0* cells is also stabilized by MCR

**Figure 8:**
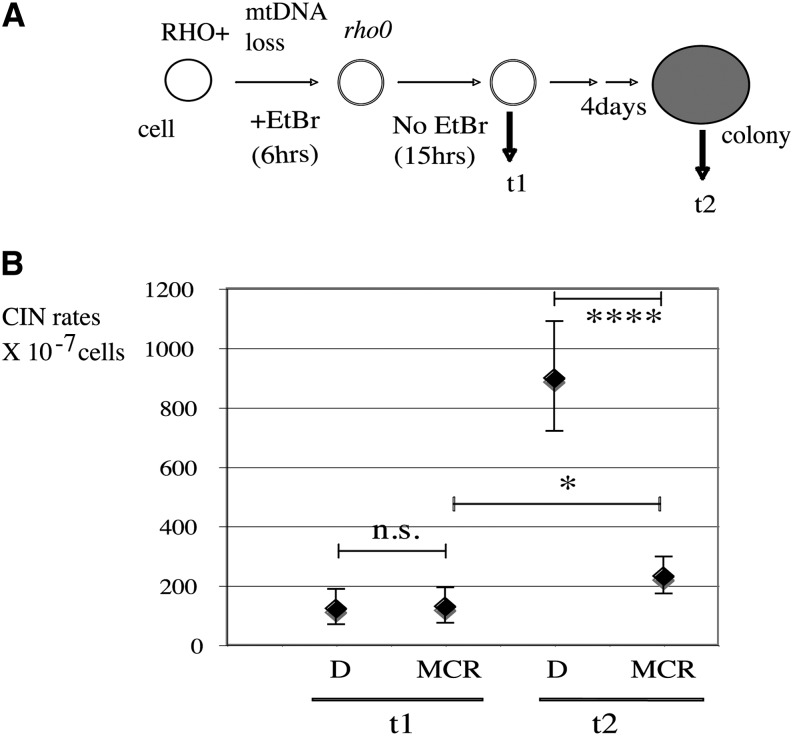
Calorie restriction stabilizes nuclear genome in newly born *rho0* cells. (A) Experimental scheme; mtDNA loss was induced in wild-type (WT) cells using ethidium bromide treatment for 6 hr and samples were taken for CINA either early after recovery (t1 = 15 hr) or later after colony growth on plate (t2 = 4 days) as described (Veatch *et al.* 2009). After ethidium bromide treatment, the experiment was performed either on regular YEPD 2% glucose (D) or under moderate calorie restriction (MCR) YEP 0.5% glucose medium. (B) Comparison of samples as explained in (A), using CINA, starting from a *RHO^+^* WT strain (L1937).

Previous experiments in this report were performed on established *rho0* cells (*i.e.*, many generations after the loss of mtDNA). To assess whether the same observations apply to newborn *rho0* cells, we repeated our experiments in cells immediately after mtDNA loss during a period termed "crisis," which is known to be prone to high CIN in *rho0* cells (Veatch *et al.* 2009). In a first step, mtDNA loss was induced in wild-type cells by growth in the presence of ethidium bromide, followed by recovery in either rich YEPD liquid medium or under MCR for 15 hr (t1) (Figure 8A). Thereafter, single cells were micro-manipulated on plates and allowed to grow to colonies for 4 d under the same conditions as during the liquid growth (D or MCR; t2) and then used for CINA. First, we detected no increase in nuclear chromosome instability in the first hours after the loss of mtDNA (t1) compared to the untreated *RHO*^+^ control (Figure 8B) (t1, D). When single cells from these cultures were allowed to grow to colonies and were retested for CIN, chromosome instability was readily detected in *rho0* cells grown on YEPD (2%) (Figure 8B) (t2, D) to the same extent as in the established slow-growing *rho0 (s)* (Figure 2) (L1993 and L2232). Second, when recovery and colony growth were performed under a MCR regimen after mtDNA loss, it drastically reduced CIN (eight-fold) (Figure 8B) (t2, MCR), just like in established *rho0 (s)* under MCR (Figure 4C) (*P* = 0.20, not significant). Thus, it appears that newborn *rho0* cells also undergo conditional instability, like the long-established *rho0* clones described.

### Unlike in *rho0* cells, CIN is constitutive in wild-type, cell-cycle, and respiratory *RHO*^+^ mutants

Our results clearly indicate that CIN in *rho0* strongly depends on the environment of the cell. To test whether other mutants with unstable genomes but still in possession of their mitochondrial DNA behave likewise, we measured CIN in a few selected *RHO*^+^ cell-cycle mutants grown under various conditions shown to affect *rho0* cells (MCR, in the presence of ethanol or antioxidant Tiron). Those included deletion mutants in the *SIC1* (p27KIP1 ortholog), *WHI5* (yeast Rb), and *MRC1* (Claspin) genes. The tested cell-cycle mutants had a general trend of constitutive CIN rates (Figure S8) very different from the environment-modulated genome instability seen in *rho0* cells. Wild-type cells showed a very mild yet significant increase in CIN after growth under MCR (*P* = 0.0019), as well as in the presence of ethanol (*P* < 0.0001) or Tiron (*P* = 0.0002) (Figure S8). Thus, the highly variable CIN rates in *rho0* strains are a characteristic not shared by *RHO*^+^ cells.

To see if mutants with mitochondrial dysfunctions but still in possession of their mtDNA also display a high conditional CIN phenotype, like in *rho0* cells, we examined the behavior of individual mutants in the respiratory chain as well as in the F1 and FO subunits of ATP synthase. In all instances, the single mutants analyzed showed a moderate increase in nuclear genome instability (two-fold to three-fold increase more than wild-type) and, contrary to *rho0* cells, showed no major fluctuation of the CIN rates in changing environments (Table S2). Thus, a simple loss of ATP production from the mitochondria is not sufficient to induce a high CIN phenotype, as previously found for other respiratory mutants (Veatch *et al.* 2009). Importantly, none of the respiratory mutants still in possession of their mitochondrial DNA had a clear conditional CIN phenotype. Thus, so far, the strong conditional CIN phenotype is a unique feature of *rho0* cells.

### An increase in pH protects from nuclear CIN in *rho0* cells

**Figure 9:**
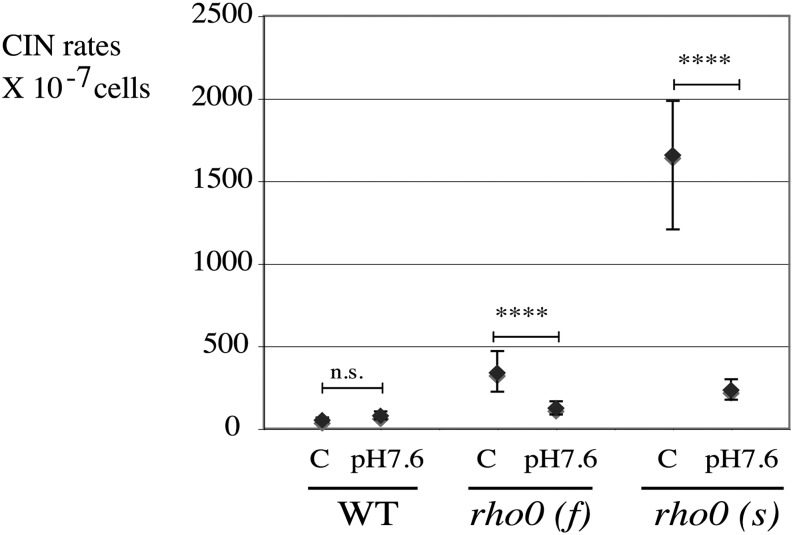
Alkaline growth pH stabilizes the nuclear genome of both fast (L2249) and slow (L2232) *rho0* cells. Comparison of control (C) YEPD medium (not buffered) and YEPD (pH = 7.6) medium buffered with 50 mM potassium phosphate buffer.

Disruption of vacuolar biogenesis or alkalinization of the yeast vacuole (that can be obtained by growth on alkaline medium) improves mitochondrial protein import, a sign of increased mitochondrial ΔΨ, through an unknown mechanism (Garipler and Dunn 2013). Because increased membrane potential is known to stabilize the nuclear genome of newborn *rho0* cells (Veatch *et al.* 2009), medium with an alkaline pH should stabilize the genome of *rho0* cells. We tested this hypothesis on wild-type, slow, and fast *rho0* cells grown on rich YEPD medium buffered at high pH (pH = 7.6, with 50 mM potassium phosphate) and measured their nuclear CIN. Nuclear chromosome instability in *rho0* cells decreased significantly (*P* < 0.0001) in both slow and fast *rho0* cells, but not in wild-type cells (*P* = 0.26, not significant), grown in alkaline conditions (Figure 9). Environmental conditions other than medium alkalinization might stabilize the nuclear genome of *rho0* cells in similar manner, *i.e.*, through increased mitochondrial ΔΨ. In mutants known as “petite-negative,” loss of mitochondrial DNA is a lethal event (Bulder 1964; Chen and Clark-Walker 2000) because of the incapacity of the cell to maintain a viable level of mitochondrial membrane potential. Thus, rescue of the petite-negative phenotype is a potential tool to screen for conditions that can improve mitochondrial ΔΨ. Mutant for the *MGR1* gene (encoding a mitochondrial i-AAA protease subunit) is one such petite-negative strain (Dunn *et al.* 2006) with lethality after mitochondrial DNA loss that can be rescued by increased vacuolar pH (Garipler and Dunn 2013). We therefore tested whether some environmental conditions, which stabilize CIN in *rho0* cells, could also rescue petite-negativity of *mgr1Δ* cells. We found that MCR improves colony growth of *mgr1* mutants after mitochondrial DNA loss, although not quite as efficiently as alkaline-buffered medium (KPi, pH 7.6) (Figure S9). Neither antioxidants nor growth at 25° rescued the growth defect of *mgr1Δ rho0* cells (data not shown). This suggests that calorie restriction might, at least in part, stabilize the genome of *rho0* cells through a mechanism involving the vacuole and the mitochondrial membrane potential.

### Inactivation of the ISC-adaptor *MMS19* leads to a moderate CIN phenotype different from *rho0* mutants

A defect in the maturation of ISC-dependent DNA metabolism and genome integrity enzymes could be responsible for the high CIN crisis in *rho0* cells, as proposed in a previous study (Veatch *et al.* 2009). A specific branch of the ISC pathway involved in the maturation of these genome integrity enzymes was recently found to depend on the ISC adaptor Mms19 (Gari *et al.* 2012; Stehling *et al.* 2012). Taken together, these results predict that deletion of *MMS19* might lead to a high chromosome instability rate, similar to CIN in *rho0* cells and that deletion of mtDNA in an *mms19Δ* strain should not increase nuclear CIN much more.

**Figure 10:**
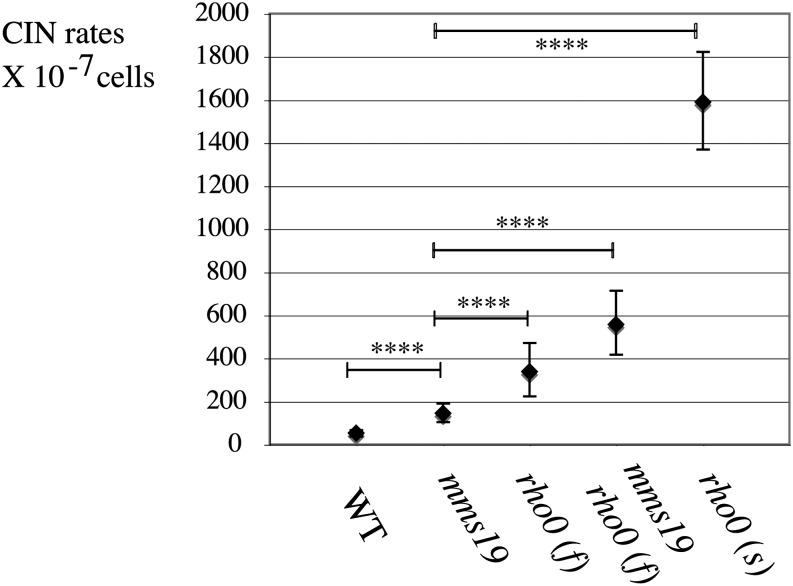
Nuclear genome instability in *rho0* cells is more severe than in the ISC-adaptor mutant *mms19*Δ. Comparison of CIN in WT (L1937), *mms19* Δ (L 2360), *rho0* (f) (L2249), *mms19* Δ *rho0* (f) (L2372), and *rho0* (s) (L2232) grown on YEPD at 30°.

**Figure 11:**
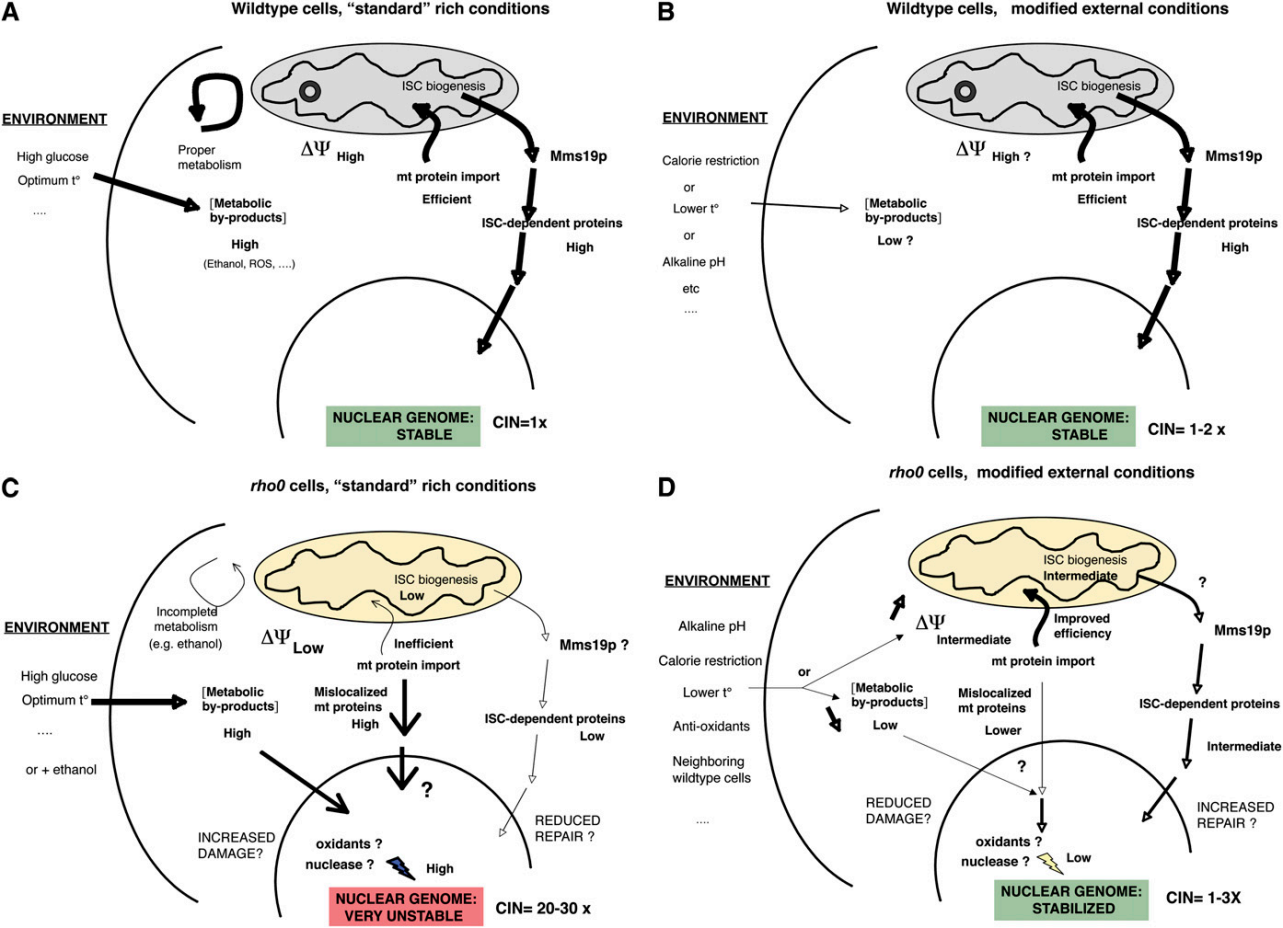
Environment-dependent nuclear genome instability in *rho0* cells. (A) Wild-type cells grown under standard/rich conditions with normal metabolic activities, including mitochondrial membrane potential ΔΨ, iron–sulfur cluster (ISC) biogenesis, and efficient import of mitochondrial protein from the cytoplasm. Metabolic byproducts can be properly processed by the cell (*e.g.*, ethanol produced from glucose can be oxidized in the mitochondria). CIN = 1X refers to the standard chromosome instability in these cells, which mainly occurs during the cell cycle. (B) In the same wild-type cells grown in different environments (calorie restriction, alkaline pH, lower t°, and others), CIN rates remain fairly constant, despite metabolic changes. (C) Cells lacking mitochondrial DNA [*rho0* (slow) or newborn *rho0* cells] grown under standard/rich conditions display very high rates of chromosome instability in postmitotic cells. Low ΔΨ leads to inefficient mitochondrial (mt) protein import and low ISC biogenesis. Metabolic byproducts like ethanol cannot be processed in the dysfunctional mitochondria. Nuclear genome instability could stem from low ISC activity (Veatch *et al.* 2009), but deletion of the ISC cofactor *MMS19* alone does not lead to high *rho0*-like instability, suggesting the existence of an additional source of nuclear CIN. An Mms19-independent branch of ISC-dependent proteins is one possibility. High genomic instability in *rho0* cells could arise from the combination of increased metabolic-dependent DNA damage (after growth in rich conditions) and defective DNA repair because of low ISC biogenesis. (D) Modified environmental conditions can fully stabilize the nuclear genome of *rho0* cells. In this model, external factors could influence nuclear CIN in *rho0* by increasing ΔΨ (like growth at alkaline pH or under moderate calorie restriction), which should increase ISC biogenesis (hence, improving DNA repair) and improve mitochondrial protein import more generally, or by decreasing metabolic byproducts (decreased DNA damage?). So far, those extreme variations of nuclear CIN in response to multiple environmental conditions are only observed in *rho0* cells.

However, we found that deletion of *MMS19* (L2360) only leads to a moderate increase in nuclear CIN (rate *mms19*Δ = 144 × 10^−7^ on YEPD, 30°) (Figure 10), approximately three-fold more than wild-type but significantly lower (*P* < 0.0001) than in any *rho0* strain tested, either fast-growing or slow-growing, and especially much lower than slow *rho0* "in crisis" [*rho0 (s)*]. We then removed mtDNA from an *mms19Δ* mutant and obtained a fast-growing *mms19 rho0* strain that showed an increased nuclear CIN compared to *mms19* alone [CIN *mms19 rho0 (f)* = 557 × 10^−7^ (L2372); *P* < 0.0001] (Figure 10). In conclusion, deletion of *MMS19* might have only a partial effect on this branch of the ISC pathway, there could be an Mms-19–independent branch of the ISC pathway defective in *rho0* cells, or, finally, additional sources of nuclear genome instability might exist in *rho0* cells. This could include an ISC-independent but ΔΨ-dependent mechanism (Figure 11).

## Discussion

Cells with mitochondrial defects are known to be prone to nuclear genome instability. Use of a highly sensitive and quantitative nuclear genome instability assay made it possible to examine the features of cells lacking mitochondrial DNA. Regarding nuclear CIN, we report here that *rho0* cells strikingly differ from most *RHO*^+^ cells in two major aspects. First, regarding timing, CIN in *rho0* cells occurs in PM cells. Second, it strongly responds to changing environments (*e.g.*, to glucose concentration or to growth temperature). This highly conditional CIN phenotype in *rho0* cells raises the question regarding how nuclear chromosomes can break in nondividing cells in response to nongenotoxic factors.

### High glucose and fast metabolism induce a genotoxic response in mitochondria-defective *rho0* cells

Glucose is essential for growth of respiratory-defective cells, which fully rely on glycolysis for ATP production. If anything, high glucose should protect nuclear genomes in *rho0* cells because glycolytic ATP is also required to maintain a mitochondrial membrane potential in *rho0* cells (Giraud and Velours 1997; Lefebvre-Legendre *et al.* 2003), a key parameter to protect nuclear genomes in those cells (Veatch *et al.* 2009). Instead, we found that high glucose concentrations lead to high nuclear genome instability in *rho0* cells. However, our data suggest that high glucose is not by itself genotoxic in *rho0* cells. Nuclear chromosomal instability occurs in PM cells, when colonies have grown and glucose is metabolized, replaced by “waste” products (such as ethanol, which cannot be used as a carbon source by mitochondria-defective cells). Secondary metabolites of glucose, rather than glucose itself, are more likely involved in this apparent genotoxicity in glucose-rich medium. Three observations are in agreement with this idea: nuclear genomes of *rho0* cells are stable during the exponential growth period, even in the presence of high glucose; addition of ethanol further destabilizes nuclear genomes in *rho0* cells; and glucose limitation (even MCR) prevents high nuclear CIN in *rho0* cells. Thus, after the growth period, a combination of secondary glucose metabolites and cell physiology (exponential *vs.* PM cells) is the likely combination that triggers high CIN in *rho0* cells. At this point, we do not know whether exponentially growing *rho0* cells are truly “protected” from nuclear chromosome damage or whether instability is not present because the CIN-inducing metabolic conditions are not met yet.

### Oxidative stress and CIN in *rho0*: a paradox

The behavior of *rho0* cells in response to oxidative stress/antioxidants is somewhat paradoxical. Our data indicate that *rho0* cells still possess efficient defenses against oxidants, including peroxides. First, nuclear CIN does not increase in the presence of "low" H_2_O_2_ (1 mM) treatment (Figure S6) [*rho0 (f*)]. Second, the main thioxiredoxin Tsa1 still protects *rho0* cells, as shown by the strong additive effects of *rho0* and *tsa1* mutations (Figure S6). Moreover, levels of endogenous H_2_O_2_ are fairly normal in *rho0* cells, whereas superoxides are very low (Delsite *et al.* 2003; Rasmussen *et al.* 2003). The hypersensitivity of *rho0* cells to peroxide (approximately 4 mM) is still unexplained and we have no evidence that it may be linked to nuclear CIN. Yet, we found that antioxidant treatment efficiently stabilizes CIN in *rho0*, but not in wild-type cells. Combined, these data suggest that chromosome instability in *rho0* may not be attributable to abnormally high peroxide levels, but rather to some deleterious event that involves an oxidant-dependent reaction. Interpreting experiments using antioxidants is difficult and is more of a hint for further experiments than an explanation by itself (Murphy *et al.* 2011). Still, genome stabilization by addition of antioxidants to *rho0* cells is clear, and this should be taken into account when proposing a mechanism for CIN.

### Potential causes for nuclear CIN in PM *rho0* cells

Chromosome breakage in unperturbed wild-type cells occurs mostly in the sensitive S-phase of the cell cycle (Lisby *et al.* 2001), with an open chromatin and active replication forks that represent a potential internal source of DNA breaks and genome instability. However, genome instability in *rho0* cells is atypical, because it occurs not in dividing but in PM cells (Figure 3). What mechanism could explain this unusual feature of CIN in resting cells? Production of high-energy radicals by *rho0* cells, leading to a direct chemical break of DNA, is one possibility. The Fenton reaction, involving the reaction of hydrogen peroxide with reduced iron Fe^2+^, could be a source of such high-energy hydroxyl radicals (Winterbourn 1995). The *rho0* cells are hypersensitive to peroxides, and the regulon controlling iron uptake is activated in *rho0* cells (Veatch *et al.* 2009); yet, we found only mild effects of modulating iron concentrations on CIN in *rho0* cells (data not shown) and inactivation of the iron regulon does not abrogate high CIN in newborn *rho0* cells (Veatch *et al.* 2009). High-energy radicals could also stem from reaction between metabolic byproducts, driven by an enzyme; in peroxiredoxin *tsa* mutants, the reaction of ethanol with hydrogen peroxide leads to high-energy radicals (1-ethoxyhydroxyl). This reaction is catalyzed by the peroxidase activity of superoxide dismutase Sod1, when this cytoplasmic enzyme is mislocalized to the nucleus (Ogusucu *et al.* 2009). In a similar way, Sod2, a nuclear-encoded mitochondrial superoxide dismutase, could potentially drive such a reaction; mislocalized Sod2 (misimported in the mitochondria because of low ΔΨ in *rho0* cells) could induce nuclear DNA damage in the presence of H_2_O_2_ and ethanol. The *rho0* cells are hypersensitive to both ethanol and H_2_O_2_ (Figure S5 and Figure S7), but we found that deletion of *SOD2* in *rho0* cells does not prevent high genomic instability (data not shown).

As previously shown, defects in mitochondrial iron–sulfur cluster biogenesis could be a main cause of CIN *in rho0* cells; decreased ISC biogenesis is sufficient to generate nuclear CIN even in *RHO*^+^ cells (Veatch *et al.* 2009). However, unlike in *rho0* cells, DNA damage induced by low ISC occurs in cycling cells (Veatch *et al.* 2009) and, unlike expected, deletion of *MMS19*, the ISC cofactor for the maturation of genome stability enzymes, only leads to a mild CIN increase, lower than CIN in *rho0* cells (Figure 10). Loss of Mms19 could only partially affect this branch of ISC biogenesis, or another Mms19-independent ISC branch could also be important for genome maintenance. Several DNA repair pathways and DNA synthesis are predicted to be affected in cells with low ISC function (Veatch *et al.* 2009). However, a repair defect does not account for the initial source of the damage. Because damage in *rho0* cells is strongly reduced in the presence antioxidants (Figure 5), oxidative-dependent damage combined with a reduced ISC-dependent DNA repair could be yet another potential source of instability in nondividing cells.

### Suppression of CIN in *rho0* cells by environmental conditions

How do external factors affect CIN in *rho0* cells? Affecting mitochondrial ΔΨ is one possibility. Increasing ΔΨ is sufficient to reduce genomic instability in *rho0* cells (Veatch *et al.* 2009). External factors that can affect mitochondrial ΔΨ are therefore expected to influence CIN in *rho0* cells. Recently, it was found that one external condition, increased pH, improves mitochondrial ΔΨ and growth properties in *rho0* cells (Garipler and Dunn 2013). Growth at alkaline pH is therefore expected to suppress CIN in *rho0* cells. We found this to be the case (Figure 9). Similarly, indirect evidence suggests that MCR might act through the same pathway (Figure S9). Thus, one category of environmental factors likely influences genomic instability in *rho0* cells through their effect on vacuolar pH and mitochondrial ΔΨ. Increased ΔΨ should improve ISC biogenesis (hence, improving DNA repair) and increase mitochondrial protein import more generally (Figure 11). We noted that the same external conditions can have opposite effects on wild-type and on *rho0* cells, because MCR prevents early alkalinization of the vacuole in wild-type cells, therefore extending lifespan (Hughes and Gottschling 2012). It was also concluded that increased vacuolar pH has an opposite effect on *RHO*^+^ and on *rho0* cells (Garipler and Dunn 2013). Our data suggest that external conditions could also influence genomic instability in *rho0* cells by affecting metabolic byproducts (ethanol, oxidants, and others) through an unknown mechanism. This is summarized in a speculative model (Figure 11).

### In a yeast colony and beyond

One technical feature of this study is that all chromosome instability assays are performed on colonies (see CINA). Growth within a colony on solid medium is complex and heterogeneous compared to growth of cells in liquid shaken media. A cell at the center or at the margin of a clone experiences increasingly different conditions as the colony grows. This difference will be marked after approximately 10 d, when a differentiation occurs (triggered by ammonia signaling in yeast) and cells at the colony center undergo apoptosis (Palkova and Vachova 2006). In our study, we distinguished between early time points (less than 42 hr), equivalent to an exponentially growing population, when we observed low nuclear CIN and an intermediate stage at 4 d, when most cells are PM, but the differentiation step described has not yet taken place; at this stage, *rho0* cells already show high nuclear CIN. This distinction is fairly crude in time and space and deserves more study. What we can state is that nuclear genome instability in *rho0* cells is not like in wild-type cells in several aspects, including timing. Yet, even though instability occurs in the PM stage in *rho0* cells, these cells are still able to resume proliferation on return to growth, because they are capable of generating CanFoA-resistant clones (in our CINA) or colony sectors (Veatch *et al.* 2009). As a colony grows, a decrease in nutrients and appearance of metabolites in the medium are likely to influence chromosome stability in *rho0* cells. The capacity of wild-type colonies to stabilize the genome of nearby *rho0* clones gives weight to this hypothesis (Figure 7); fast-growing wild-type colonies likely generate a calorie restriction zone for *rho0* cells (a CIN-stabilizing condition) and could deplete metabolites like ethanol (CIN-destabilizing), a reaction that *rho0* cells are unable to perform.

If our observations apply to metazoans, then liver would seem like one place where such effects may occur because most liver cells are nondividing and highly exposed to incoming nutrients (glucose, ethanol, or others). Under specific nutrient conditions, this could lead to genotypic changes, including loss of heterozygosity in cells lacking mtDNA. Although cancer cells often display mutations in mitochondrial genes, the depletion of mtDNA from cancer cells reduced tumorigenicity in a few tested instances (Cavalli *et al.* 1997; Morais *et al.* 1994; Wallace 2012). It is not clear whether *rho0* cells and their dysfunctions could be a source of disease. Because loss of mitochondrial DNA increases with aging (Veatch *et al.* 2009), and because high nuclear genomic instability occurs in nonproliferating *rho0* cells, PM cells stemming from aged mother cells could be affected.

## Supplementary Material

Supporting Information
