# Supplementary material for: Metabolic and Environmental Conditions Determine Nuclear Genomic Instability in Budding Yeast Lacking Mitochondrial DNA
Source: G3 (Bethesda). 2013 Dec 27;4(3):411–23. doi: 10.1534/g3.113.010108 (PMC3962481; doi:10.1534/g3.113.010108)
Supplement: Supporting Information [file supp_4_3_411__index.html]

Metabolic and Environmental Conditions Determine Nuclear Genomic Instability in Budding Yeast Lacking Mitochondrial DNA — Supporting Information 

# Metabolic and Environmental Conditions Determine Nuclear Genomic Instability in Budding Yeast Lacking Mitochondrial DNA

## Supporting Information for Dirick *et al.*, 2014

**Files in this Data Supplement:**

- Supporting Information - Figures S1-S10 and Tables S1-S2 (PDF, 8 MB)
- Figure S1 - Chromosome breaks and chromosome loss in wildtype cells using CINAssay. (PDF, 890 KB)
- Figure S2 - Irreversible and efficient switch from slow to fast growing *rho0* clones. (PDF, 1 MB)
- Figure S3 - Chromosome breaks are the main source of nuclear genome instability in *rho0* cells. (PDF, 1 MB)
- Figure S4 - Point mutation rates at *CAN1* locus are not elevated in cells lacking mitochondrial DNA. (PDF, 60 KB)
- Figure S5 - *rho0* cells show hypersensitivity to oxidative stress by H2O2 but not to tested cell cycle or DNA damage drugs. (PDF, 2 MB)
- Figure S6 - (A) Highly unstable nuclear genomes in *rho0* cells lacking the peroxiredoxin gene *TSA1*. (B) CIN in *tsa1 RHO*+ strain (L1822) is high under standard conditions (D=YEPD 30°) and is not reduced by calorie restriction nor by low growth temperature (25°), unlike in *rho0* cells. (PDF, 68 KB)
- Figure S7 - Ethanol slows down *rho0* colony growth but does not select for suppressors. (PDF, 3 MB)
- Figure S8 - Unlike in *rho0* cells, CIN is fairly constitutive in wildtype and cell cycle mutants grown in various environmental conditions. (PDF, 67 KB)
- Figure S9 - Suppression of the petite-negative *mgr1* mutant by moderate calorie restriction. (PDF, 915 KB)
- Figure S10 - Factors shown to affect instability in *rho0* cells. (PDF, 88 KB)
- Table S1 - Strains used in this study. (PDF, 127 KB)
- Table S2 - Nuclear genome instability in wildtype and in respiratory mutants under various growth conditions. (PDF, 104 KB)
