# Supplementary material for: Metabolic and Environmental Conditions Determine Nuclear Genomic Instability in Budding Yeast Lacking Mitochondrial DNA
Source: G3 (Bethesda). 2013 Dec 27;4(3):411–23. doi: 10.1534/g3.113.010108 (PMC3962481; doi:10.1534/g3.113.010108)
Supplement: Supporting Information [file supp_g3.113.010108_010108SI.pdf]

G3 · Genes | Genomes | Genetics

Metabolic and environmental conditions determine nuclear genomic instability in budding yeast lacking mitochondrial DNA

Léon DIRICK, Walid BENDRIS, Vincent LOUBIERE, Thierry GOSTAN, Elisabeth GUEYDON and Etienne SCHWOB

Institut de Génétique Moléculaire de Montpellier UMR 5535 CNRS, 1919 route de Mende, 34293 Montpellier cedex 5, France; Université Montpellier 2, Place Eugène Bataillon, 34095 Montpellier cedex 5; Université Montpellier 1, 5 Bd Henri IV, 34967 Montpellier cedex 2

Corresponding author: Léon DIRICK

Mailing address: IGMM, CNRS UMR5535, 1919 route de Mende, 34293 Montpellier cedex 5, France
Telephone : (33) 434 35 96 77
Fax : (33) 434 35 96 34
Email : leon.dirick@igmm.cnrs.fr

DOI: 10.1534/g3.113.010108

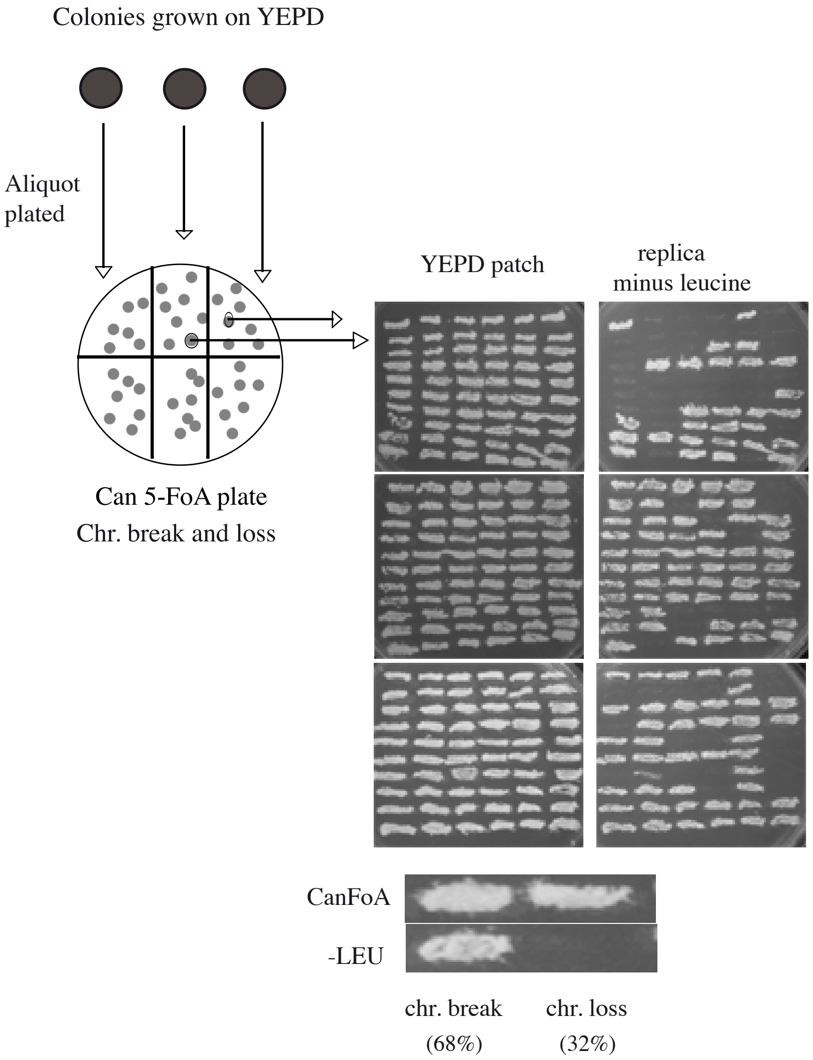

Figure S1 Chromsome breaks and chromosome loss in wildtype cells using CINAssay. Clones with chromsome instabilities were picked from Can 5-FoA plates and patched on YEPD; replica on medium lacking leucine allows to distinguish between chromosome breaks (Leu+) and chromosome loss (Leu-) (see Figure 1A). The six patches aligned horizontally represent CanFoA resistant clones randomly picked from each assay. Below, a magnified view , with the percentage of chromosome breaks (about 2/3) and loss (1/3). A more global method, which consist of replica plating the whole CanFOA plates is also useful but difficult to quantify precisely (see Figure S7C)

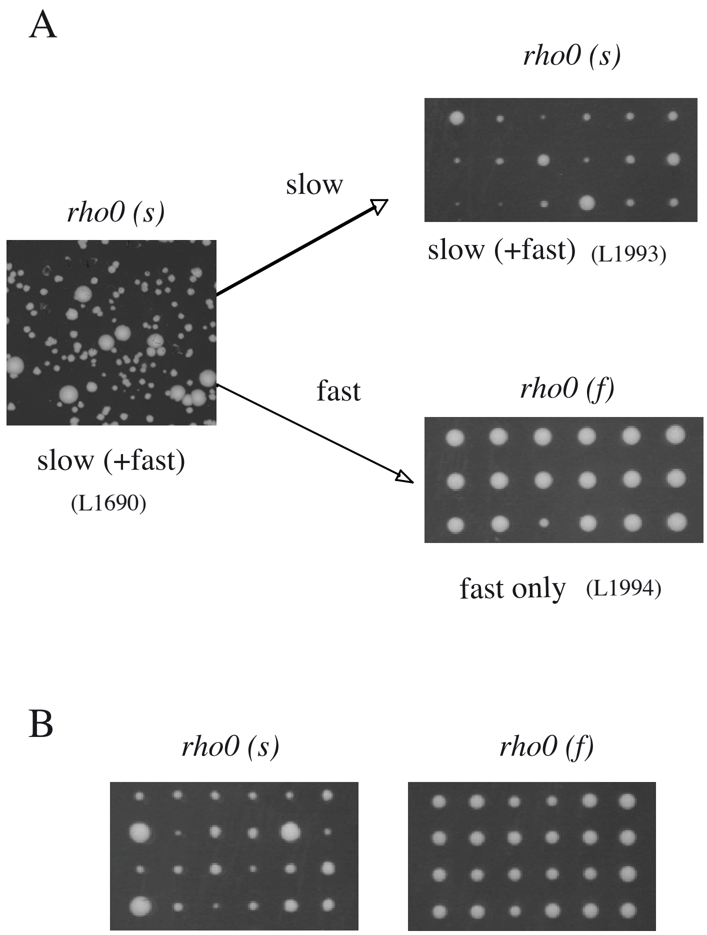

Figure S2 Irreversible and efficient switch from slow to fast growing *rho0* clones. (A) Fast growing colonies emerge among slow growing *rho0 (s)* clones (L1690, induced with ethidium bromide). *rho0 (s)* cells on YPD give rise to both slow and fast growing clones (L1993) while fast growing cells always lead to fast growing clones (L1994). (B) the same phenomenon is observed with spontaneous *rho0* cells (no ethidium bromide; *rho0 (s)* L2232 and *rho0 (f)* L2249). All cells are *rho0* and therefore can not reverse to a respiratory competent state.

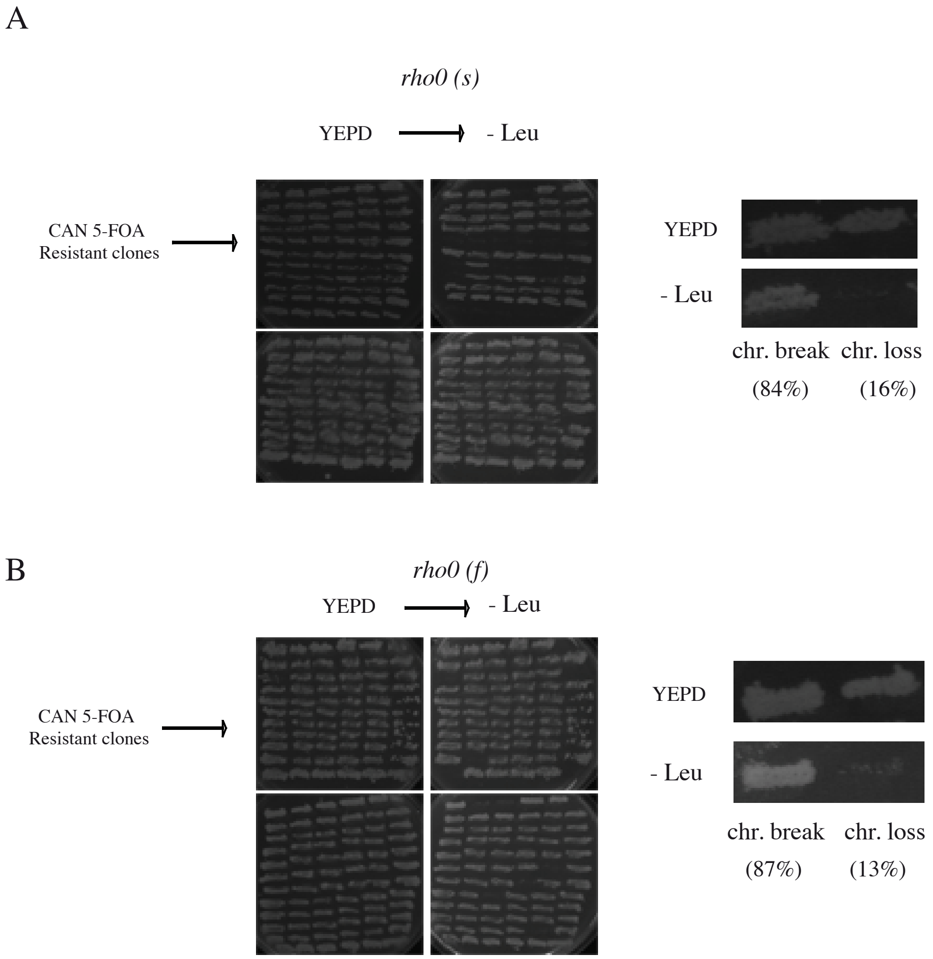

Figure S3 Chromosome breaks is the main source of nuclear genome instability in *rho0* cells. (A) *rho0 (s)* (L2232) and (B) *rho0 (f)* (L2249) were grown to colonies on YEPD at 30° for 4 days, then subjected to CINAassay (see Figure 2). Breaks and loss are distinguished as described for wildtype in Figure S1. Chromosome breaks account for over 80% of instability events in *rho0* cells.

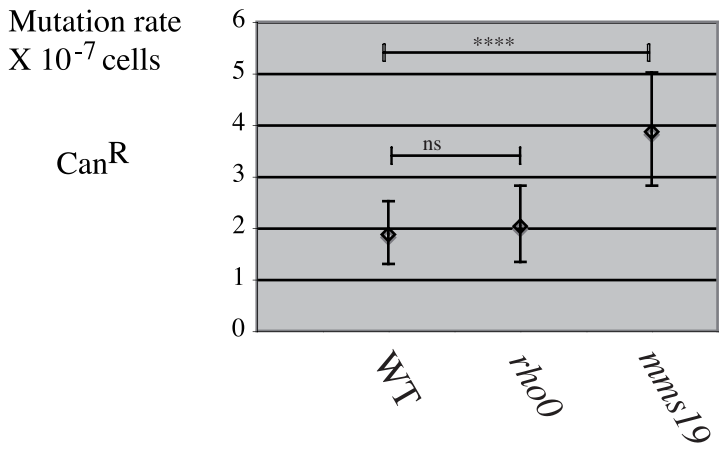

Figure S4 Point mutation rates at *CAN1* locus are not elevated in cells lacking mitochondrial DNA. Wildtype (L1459), *rho0 (f)* (L1472) and *mms19* deletion mutant (L2356) grown to colonies on YEPD at 30° for 3 days, then plated on canavanine plates.

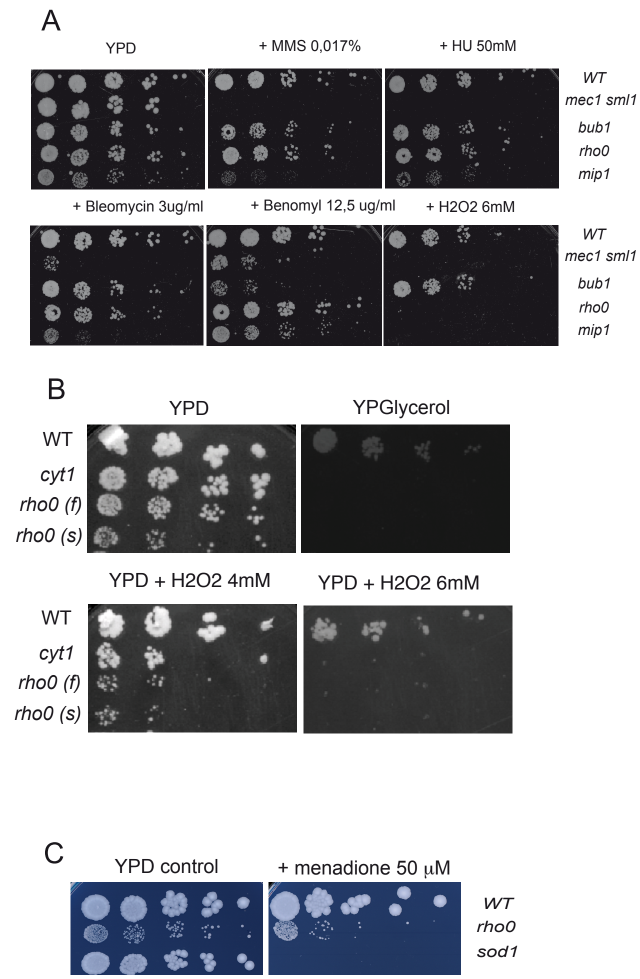

Figure S5 *rho0* cells show hypersensitivity to oxidative stress by H2O2 but not to tested cell cycle or DNA damage drugs. Drop test by serial dilution (5x) of cell suspensions (A) with indicated drugs, added to YEPD plates and insubated for 2 days at 30°. MMS (methymehanesulfonate), HU (hydroxyurea). WT (L1577), *rho0 (f)* (L1994), mitochondrial DNA polymerase mutant *mip1* (*rho0 (s)*, L 1779). Checkpoint mutant controls: *mec1 sml1* (L1734), *bub1* (L1783) (B) Drop test with WT (L1937), *cyt1 RHO+* (L1799) spontaneous *rho0 (s)* strain (L2232), *rho0 (f)* strain (L2249), on YEPD, YEPD + H2O2 and YEPGlycerol plates (C) *rho0* are not hypersentive to oxidative stress by superoxide generating agent menadione. WT (L1937), spontaneous *rho0 (s)* (L2232) and *sod1 RHO+* (L1995)

A

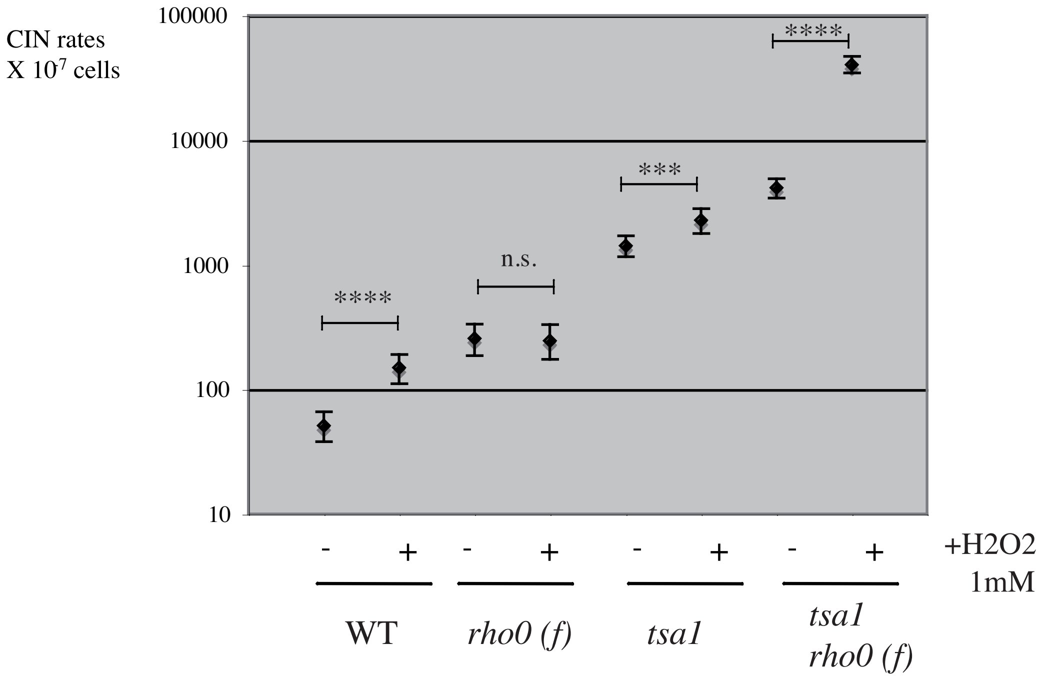

B

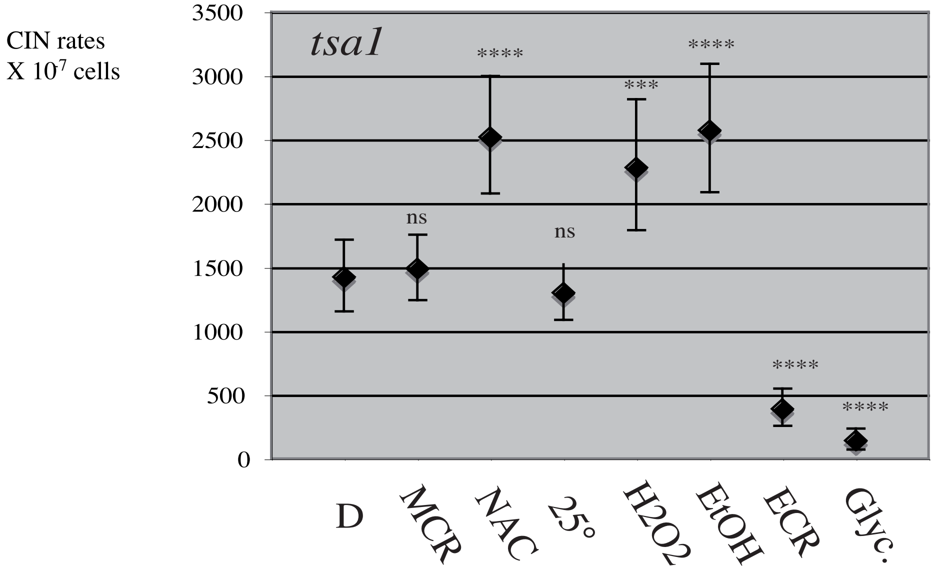

Figure S6 (A) Highly unstable nuclear genomes in *rho0* cells lacking the peroxiredoxin gene *TSA1*. WT (L1937), *rho0 (f)* (L1994) , *tsa1 RHO+* (L1822) and *tsa1 rho0 (f)* (L1829) were grown on YEPD 2% at 30°, with ot without 1mM H202, and tested by CINAassay. Note the log Y axis. (B) CIN in *tsa1 RHO+* strain (L1822) is high under standard conditions (D=YEPD 30°) and is not reduced by calorie restriction nor by low growth temperature (25°), unlike in *rho0* cells. Ethanol, peroxides and NAC induced a moderate increase of CIN in this strain (less than 2 fold). However, extreme calorie restriction (ECR= 0.05% glucose and YPGlycerol (2%)) leads to a significant stabilization of these cells (4 to 10 fold, $p<0.0001$) compared to standard YEPD 30° conditions.

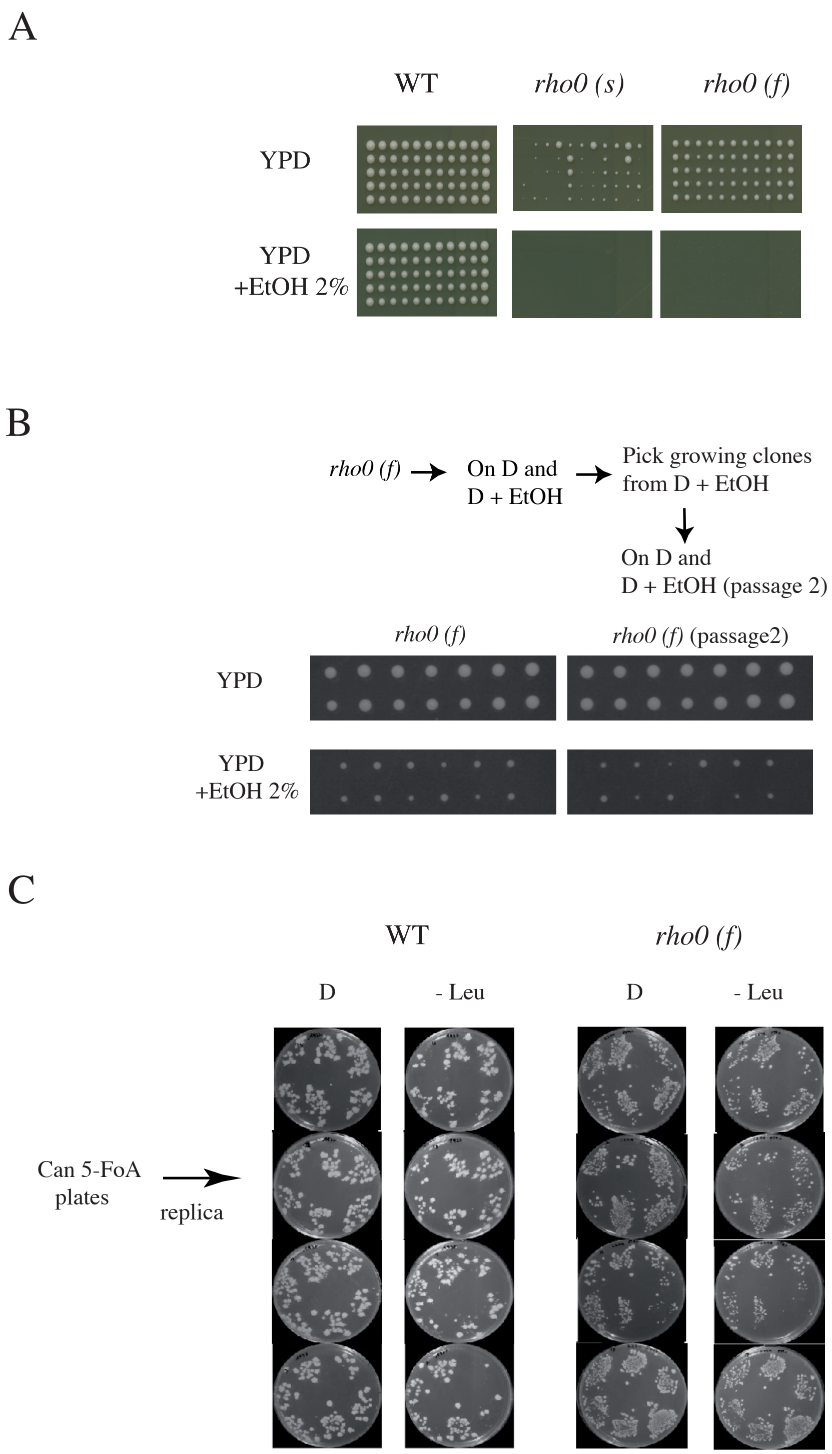

Figure S7 Ethanol slows down *rho0* colony growth but does not select for suppressors (A) Clonal growth of WT, *rho0 (s)* and *rho0 (f)* CINA strains on YEPD and YEPD + ethanol 2% at 30° for 3 days. (B) One day later, *rho0 (f)* colonies become visible on YEPD+ ethanol and can be tested for CINA (see Figure 6). Individual cells from this plate do not show faster growth when tested again on YEPD + ethanol 2 % (passage 2) , indicating that they are not suppressors. (C) Chromosome breaks and loss in cells grown in the presence of ethanol. WT (L1937) and *rho0 (f)* (L2249) were grown to colonies on YEPD + 2% ethanol and tested by CINA (see Figure 6), then replica plated on - Leu plates to estimate the relative frequency of chromosome breaks (LEU+) versus loss (LEU-).

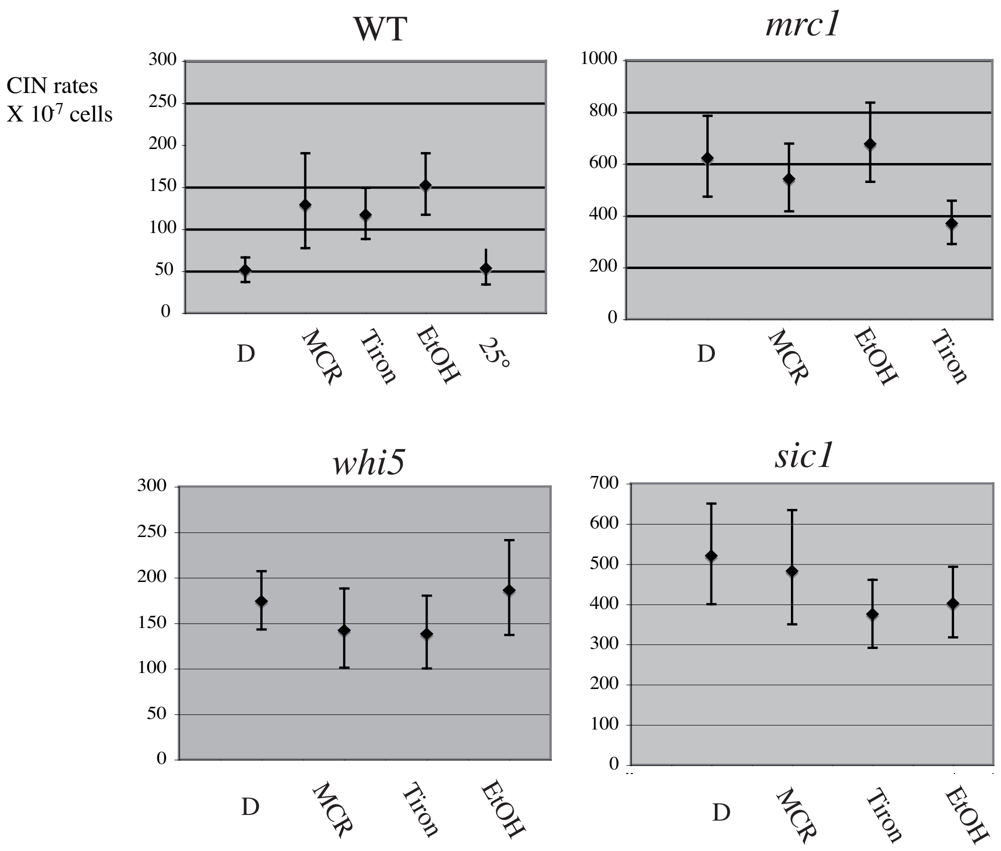

Figure S8 Unlike in *rho0* cells, CIN is fairly constitutive in wildtype and cell cycle mutants grown in various environmental conditions. CINassays were performed on cells deleted for the RB-homolog *WHI5* (*whi5*, L1888), the yeast Claspin homolog *MRC1* (*mrc1*, L1925) and the Cdk/CyclinB inhibitor *SIC1* (*sic1*, L1580). Colonies were grown on plates at 30°. D= YEPD; MCR=moderate calorie restriction (YEP+ 0,5% glucose); Tiron= YEPD + Tiron 1mM ; EtOH= YEPD + 2% ethanol.

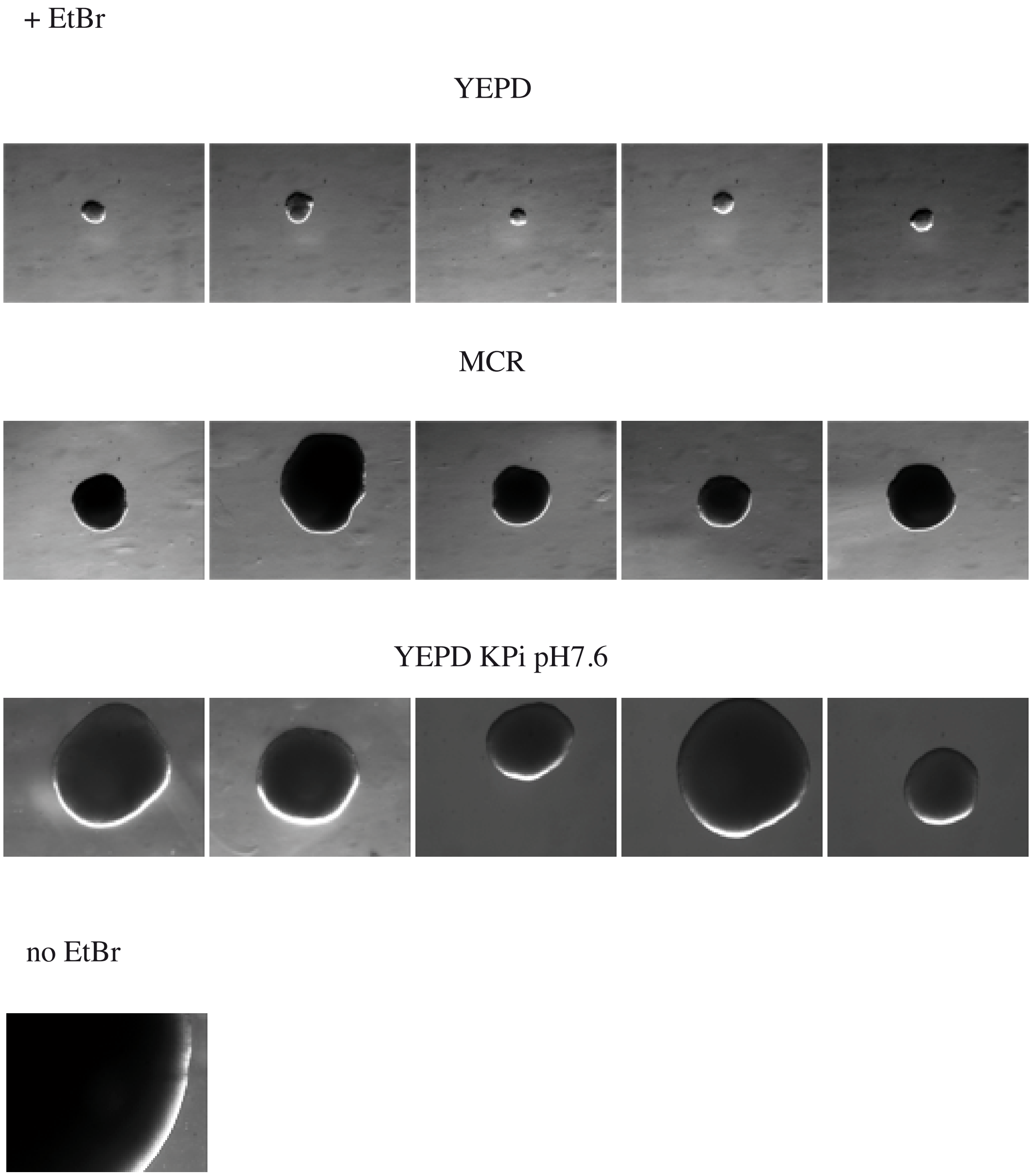

Figure S9 Suppression of the petite-negative *mgr1* mutant by moderate calorie restriction. *mgr1*Δ *RHO+* strain (L 2407) was subjected to ethidium bromide (+EtBr) to induce the loss of mtDNA, and single cells were micromanipulated on unbuffered rich medium (YEPD), under moderate calorie restriction conditions (MCR) or on rich medium buffered at alkaline pH (KPi, pH 7.6), as a positive control. Colony growth at day 4 , 30°. "no EtBr"represents the edge of an untreated *mgr1 RHO+* cell on YEPD. Pictures were taken under light microscope, 10x magnification.

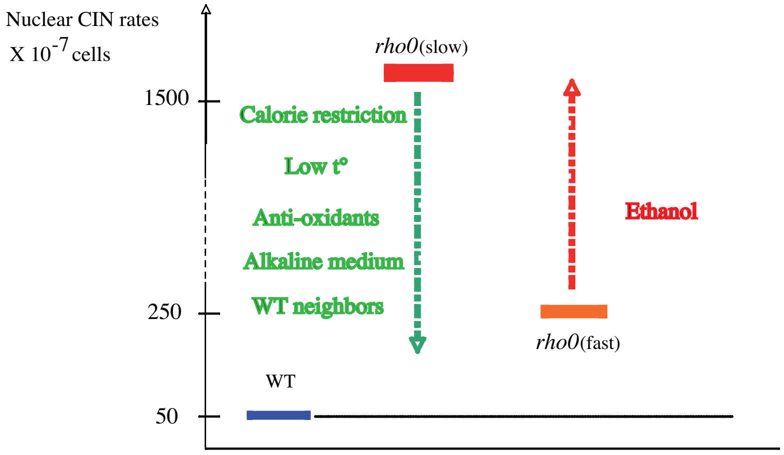

Figure S10 Factors shown to affect instability in *rho0* cells. Bars for WT, slow *rho0 (s)* and fast *rho0 (f)* indicate nuclear CIN in those strains under standard rich conditions (YEPD 30°). Factors in green can stabilize the genome of a *rho0 (s)* close to WT levels. In the presence of ethanol (2%), a moderately unstable *rho0 (f)* strain becomes as unstable as a *rho0 (s)*. Some factors might influence CIN through their effect on mitochondrial membrane potential (affecting ISC-dependent DNA repair), others by their effect on metabolic byproducts (affecting DNA damage?) (see Figure 11).

Table S1 Strains used in this study

Strain # (L)	Genotype	Origin/Note
1459	*MATa, ura3-52, leu2Δ1, trp1Δ63, his3Δ200, lys2ΔBgl, hom3-10, ade2Δ1, ade8, hxt13::URA3 CAN1*	RDKY3615 (S288C) (CHEN and KOLODNER 1999)
1472	*MATa, ura3-52, leu2Δ1, trp1Δ63, his3Δ200, lys2ΔBgl, hom3-10, ade2Δ1, ade8, hxt13::URA3 rho0*	1459 +ethidium bromide
1520	*MATa, leu2Δ1, trp1Δ63, his3Δ200, lys2ΔBgl, hom3-10, ade2Δ1, ade8, hxt13::URA3 CAN1 ura3-52::LEU2*	1459 with integrated ura3-52::LEU2 (pUL9, F. Cross)
1571	*MATalpha, his3 ::1 leu2::0 met15::0 ura3::0 can1::kanMX 4*	S288C (BY, Euroscarf)
1577	*MATa/alpha ura3-52::LEU2, leu2Δ1, trp1Δ63, his3Δ200, lys2ΔBgl, hom3-10, ade2Δ1, ade8, CAN1, hxt13::URA3/ his3 ::1 leu2::0 met15::0 ura3::0 can1::kanMX 4*	1520 x 1571 wild type; CINA strain
1580	*MATa/alpha his3 ::1 leu2::0 met15::0 ura3::0 sic1::kanMX 4 can1::kanMX 4/,ura3-52::LEU2, leu2Δ1, trp1Δ63, his3Δ200, lys2ΔBgl, hom3-10 ade2Δ1, ade8, hxt13::URA3 sic1::HIS3*	*sic1 Δ RHO+*; CINA strain
1607	*MATalpha leu2::0 lys2::0 met15::0 trp1::63 ura3::0 can1*	S288c (BY4727); this study
1690	*MATa/alpha ura3-52::LEU2, leu2Δ1, trp1Δ63, his3Δ200, lys2ΔBgl, hom3-10, ade2Δ1, ade8, CAN1, hxt13::URA3/ his3 ::1 leu2::0 met15::0 ura3::0 can1::kanMX 4 rho0*	*rho0* (s); CINA strain 1577 +ethidium bromide; Slow growing
1779	*MATa/alpha his3 ::1 leu2::0 met15::0 ura3::0 trp1 mip1::kanMX4 hxt13::URA3 CAN1 / his3 ::1 leu2::0 ura3::0 can1::kanMX 4 mip1::kanMX4 rho0*	*mip1 Δ rho0 (s)*; CINA strain
1783	*MATa his3 ::1 leu2::0 met15::0 ura3::0 bub1::kanMX4*	*bub1 Δ* ;S288C (BY, Euroscarf)
1799	*MATa/alpha, cyt1::KanMX ura3-52::LEU2 hxt13::URA3 CAN1 (S288c) omnc/ his3 ::1 leu2::0 met15::0 ura3::0 can1 cyt1::kanMX4*	*cyt1 Δ RHO+*; CINA strain
1801	*MATa/alpha his3 ::1 leu2::0 met15::0 ura3::0 can1 clb5::kanMX4/ ura3-52::LEU2 hxt13::URA3 CAN1 clb5::kanMX4*	*clb5 Δ*; CINA strain
1822	*MATa/alpha his3 ::1 leu2::0 met15::0 ura3::0 tsa1::kanMX 4 ura3-52::LEU2 hxt13::URA3 CAN1/ his3 ::1 leu2::0 met15::0 ura3::0 tsa1::kanMX 4 can1*	*tsa1 Δ*; CINA strain
1829	*MATa/alpha his3 ::1 leu2::0 met15::0 ura3::0 tsa1::kanMX 4 ura3-52::LEU2 hxt13::URA3 CAN1/ his3 ::1 leu2::0 met15::0 ura3::0 tsa1::kanMX 4 can1 rho0*	*tsa1 Δ rho0*; CINA strain
1847	*MATalpha ura3-52::LEU2 hxt13::URA3 CAN1 his3 TRP+*	S288C; 1577 spo.
1888	*MATa/alpha his3 ::1 leu2::0 met15::0 ura3::0 whi5::kanMX 4 ura3-52::LEU2 hxt13::URA3 CAN1/whi5::kanMX 4 can1*	*whi5 Δ RHO+;* CINA strain
1925	*MATa/alpha, ura3-52::LEU2 hxt13::URA3 CAN1 his3 mrc1::kanMX4 TRP+ /leu2::0 lys2::0 met15::0 trp1::63 ura3::0 can1 mrc1::kanMX4*	*mrc1 Δ RHO+;* CINA strain

1937	*MATa/alpha ura3-52::LEU2, leu2Δ1, trp1Δ63, his3Δ200, lys2ΔBgl, hom3-10, ade2Δ1, ade8, CAN1, hxt13::URA3/ his3 ::1 leu2::0 met15::0 ura3::0 can1::kanMX 4*	1577; subclone. Wildtype CINA strain
1993	*MATa/alpha ura3-52::LEU2, leu2Δ1, trp1Δ63, his3Δ200, lys2ΔBgl, hom3-10, ade2Δ1, ade8, CAN1, hxt13::URA3/ his3 ::1 leu2::0 met15::0 ura3::0 can1::kanMX 4 rho0 (s)*	Subclone of 1690; slow growing rho0; CINA strain
1994	*MATa/alpha ura3-52::LEU2, leu2Δ1, trp1Δ63, his3Δ200, lys2ΔBgl, hom3-10, ade2Δ1, ade8, CAN1, hxt13::URA3/ his3 ::1 leu2::0 met15::0 ura3::0 can1::kanMX 4 rho0 (f)*	Subclone of 1690; fast growing rho0; CINA strain
2232	*MATa/alpha, ura3-52::LEU2, leu2Δ1, trp1Δ63, his3Δ200, lys2ΔBgl, hom3-10, ade2Δ1, ade8, hxt13::URA3/ his3 ::1 leu2::0 met15::0 ura3::0 can1::kanMX 4 rho0 (s)*	Spontaneous *rho0* (slow) isolated from 1937; CINA strain
2249	*MATa/alpha, ura3-52::LEU2, leu2Δ1, trp1Δ63, his3Δ200, lys2ΔBgl, hom3-10, ade2Δ1, ade8, hxt13::URA3/ his3 ::1 leu2::0 met15::0 ura3::0 can1::kanMX 4 rho0 (f)*	Spontaneous *rho0* (fast) isolated from 2232; CINA strain
2264	*MATa/alpha his3 ::1 leu2::0 met15::0 ura3::0 atp11::KanMx4 ura3-52::LEU2 hxt13::URA3 CAN1*	*atp11Δ*; CINA strain
2267	*MATa/alpha his3 ::1 leu2::0 met15::0 ura3::0 atp10::KanMx4 ura3-52::LEU2 hxt13::URA3 CAN1/atp10::KanMx4 can1*	*atp10Δ*; CINA strain
2288	*MATa/alpha his3 ::1 leu2::0 met15::0 ura3::0 sΔh4::KanMx4 ura3-52::LEU2 hxt13::URA3 CAN1/his3 ::1 leu2::0 met15::0 ura3::0 sdh4::KanMx4 can1*	*sdh4Δ*; CINA strain
2360	*MATa/alpha, ura3-52::LEU2 hxt13::URA3 CAN1 his3 mms19::KanMx4 TRP+ / can1 mms19::KanMx4*	*mms19 Δ*; CINA strain
2372	*MATa/alpha, ura3-52::LEU2 hxt13::URA3 CAN1 his3 mms19::KanMx4 TRP+ / can1 mms19::KanMx4 rho0*	2360 ; *rho0* (fast); CINA strain
2407	*MATa his3 ::1 leu2::0 met15::0 ura3::0 mgr1::kanMX4*	*mgr1 Δ* ; S288C (BY, Euroscarf)

Table S2 Nuclear genome instability in wildtype and in respiratory mutants under various growth conditions

genotype	Complex affected	CIN D 30°	CIN D 25°	CIN M.C.R. (30°)	CIN D +EtOH (30°)	Strain# L
wildtype	/	5 ± 2	6 ± 1	12 ± 1	13 ± 2	1937
rho0 slow	RC + F0	145 ± 14	8 ± 1	17 ± 2	NV	1993
rho0 fast	RC + F0	43 ± 14	6 ± 1	18 ± 7	129 ± 6	1994
cyt1	RC III	15 ± 11	24 ± 4	13 ± 3	14 ± 1	1799
sdh4	RC II	19 ± 2	ND	ND	ND	2288
atp10	RC V (F0)	12 ± 1	7 ± 1	8 ± 2	14 ± 1	2267
atp11	RC V (F1)	20 ± 4	ND	15 ± 5	20 ± 3	2264

CIN rates (x 10^{-6} viable cells, Lea and Coulson Method of the Median) RC = respiratory chain complex ; ND= Not Determined ; NV= Not Viable. Conditions : YPD (2%) D 30° and 25° ; MCR, moderate calorie restriction (0,5% D) ; D + EtOH (YPD + 2% ethanol) ; FO/F1= F0/F1 subunit of ATPsynthase/-ase (RC V); *rho0* cells are mutant in RC III-IV and in FO of RC V
