# Supplementary material for: Metabolic and Environmental Conditions Determine Nuclear Genomic Instability in Budding Yeast Lacking Mitochondrial DNA
Source: G3 (Bethesda). 2013 Dec 27;4(3):411–23. doi: 10.1534/g3.113.010108 (PMC3962481; doi:10.1534/g3.113.010108)
Supplement: Supporting Information [file supp_g3.113.010108_FigureS1.pdf]

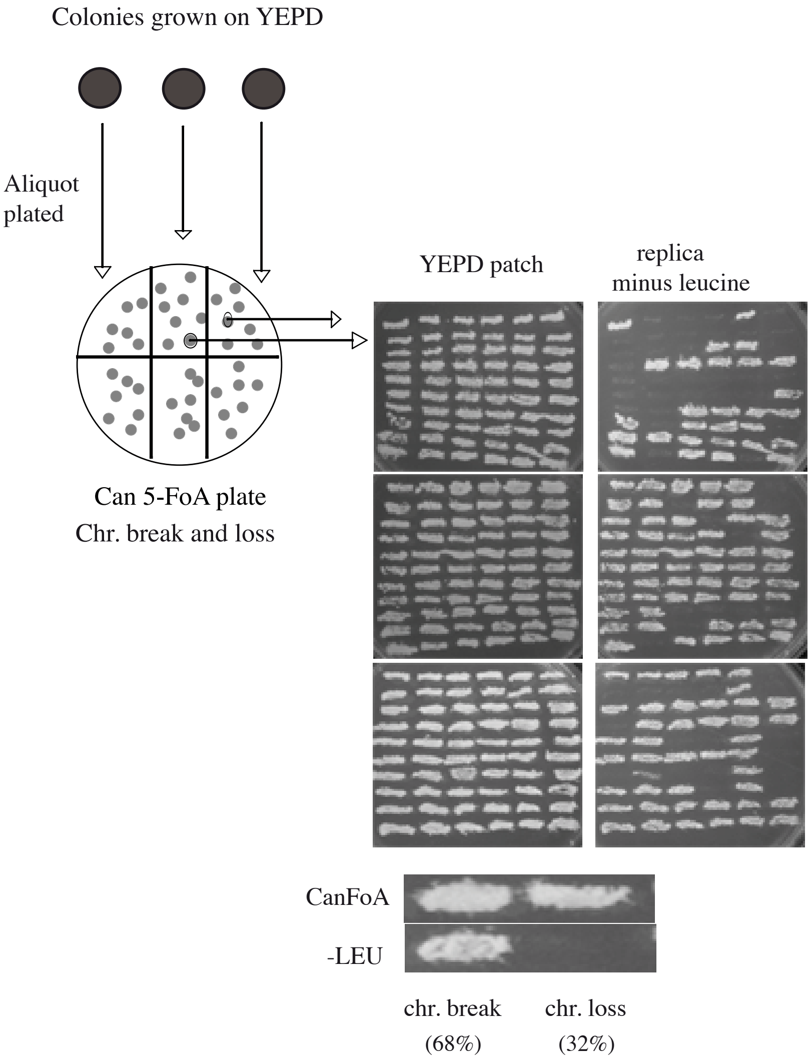


**Figure S1** Chromsome breaks and chromosome loss in wildtype cells using CINAssay. Clones with chromsome instabilities were picked from Can 5-FoA plates and patched on YEPD; replica on medium lacking leucine allows to distinguish between chromosome breaks (Leu+ ) and chromosome loss (Leu-) (see Figure 1A). The six patches aligned horizontally represent CanFoA resistant clones randomly picked from each assay. Below, a magnified view , with the percentage of chromosome breaks (about 2/3) and loss (1/3). A more global method, which consist of replica plating the whole CanFOA plates is also useful but difficult to quantify precisely (see Figure S7C)
