# Supplementary material for: Metabolic and Environmental Conditions Determine Nuclear Genomic Instability in Budding Yeast Lacking Mitochondrial DNA
Source: G3 (Bethesda). 2013 Dec 27;4(3):411–23. doi: 10.1534/g3.113.010108 (PMC3962481; doi:10.1534/g3.113.010108)
Supplement: Supporting Information [file supp_g3.113.010108_FigureS2.pdf]

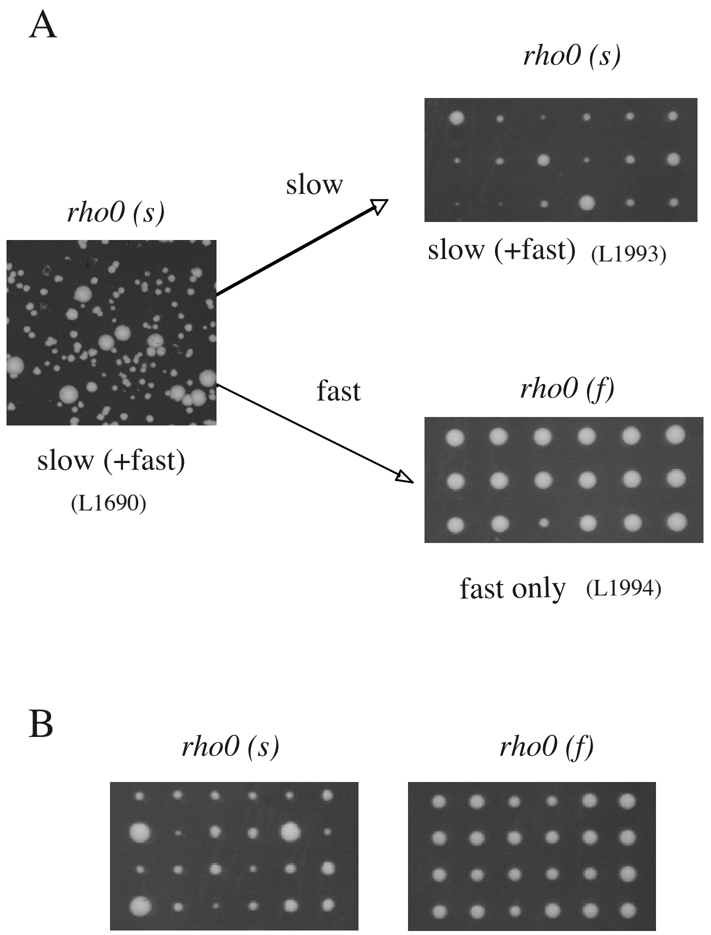

Figure S2 Irreversible and efficient switch from slow to fast growing *rho0* clones. (A) Fast growing colonies emerge among slow growing *rho0 (s)* clones (L1690, induced with ethidium bromide). *rho0 (s)* cells on YPD give rise to both slow and fast growing clones (L1993) while fast growing cells always lead to fast growing clones (L1994). (B) the same phenomenon is observed with spontaneous *rho0* cells (no ethidium bromide; *rho0 (s)* L2232 and *rho0 (f)* L2249). All cells are *rho0* and therefore can not reverse to a respiratory competent state.
