# Supplementary material for: Metabolic and Environmental Conditions Determine Nuclear Genomic Instability in Budding Yeast Lacking Mitochondrial DNA
Source: G3 (Bethesda). 2013 Dec 27;4(3):411–23. doi: 10.1534/g3.113.010108 (PMC3962481; doi:10.1534/g3.113.010108)
Supplement: Supporting Information [file supp_g3.113.010108_FigureS3.pdf]

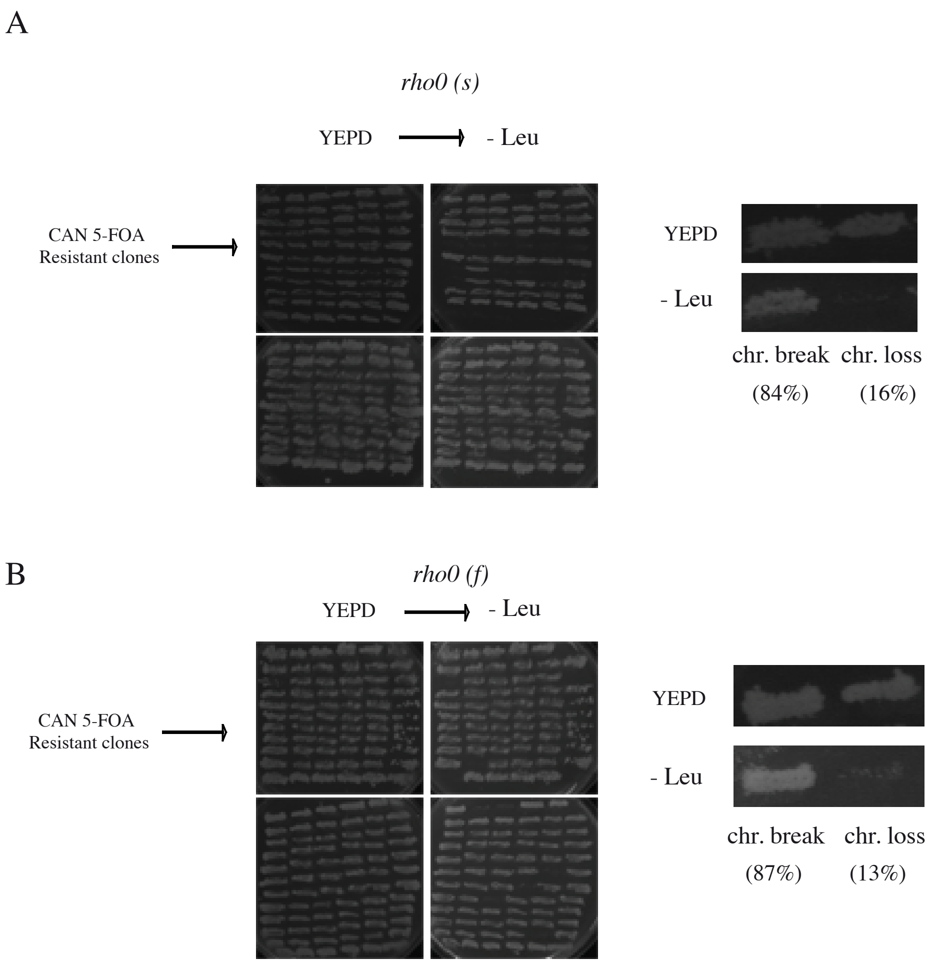

Figure S3 Chromosome breaks is the main source of nuclear genome instability in *rho0* cells. (A) *rho0 (s)* (L2232) and (B) *rho0 (f)* (L2249) were grown to colonies on YEPD at 30° for 4 days, then subjected to CINAassay (see Figure 2). Breaks and loss are distinguished as described for wildtype in Figure S1. Chromosome breaks account for over 80% of instability events in *rho0* cells.
