# Supplementary material for: Metabolic and Environmental Conditions Determine Nuclear Genomic Instability in Budding Yeast Lacking Mitochondrial DNA
Source: G3 (Bethesda). 2013 Dec 27;4(3):411–23. doi: 10.1534/g3.113.010108 (PMC3962481; doi:10.1534/g3.113.010108)
Supplement: Supporting Information [file supp_g3.113.010108_FigureS4.pdf]

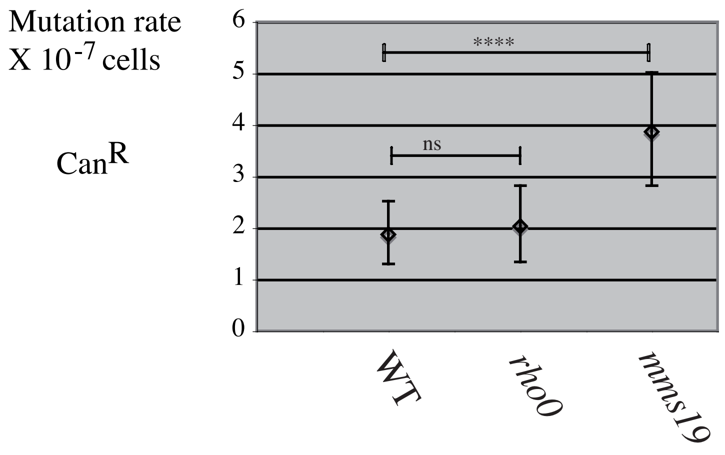

Figure S4 Point mutation rates at *CAN1* locus are not elevated in cells lacking mitochondrial DNA. Wildtype (L1459), *rho0 (f)* (L1472) and *mms19* deletion mutant (L2356) grown to colonies on YEPD at 30° for 3 days, then plated on canavanine plates.
