# Supplementary material for: Metabolic and Environmental Conditions Determine Nuclear Genomic Instability in Budding Yeast Lacking Mitochondrial DNA
Source: G3 (Bethesda). 2013 Dec 27;4(3):411–23. doi: 10.1534/g3.113.010108 (PMC3962481; doi:10.1534/g3.113.010108)
Supplement: Supporting Information [file supp_g3.113.010108_FigureS5.pdf]

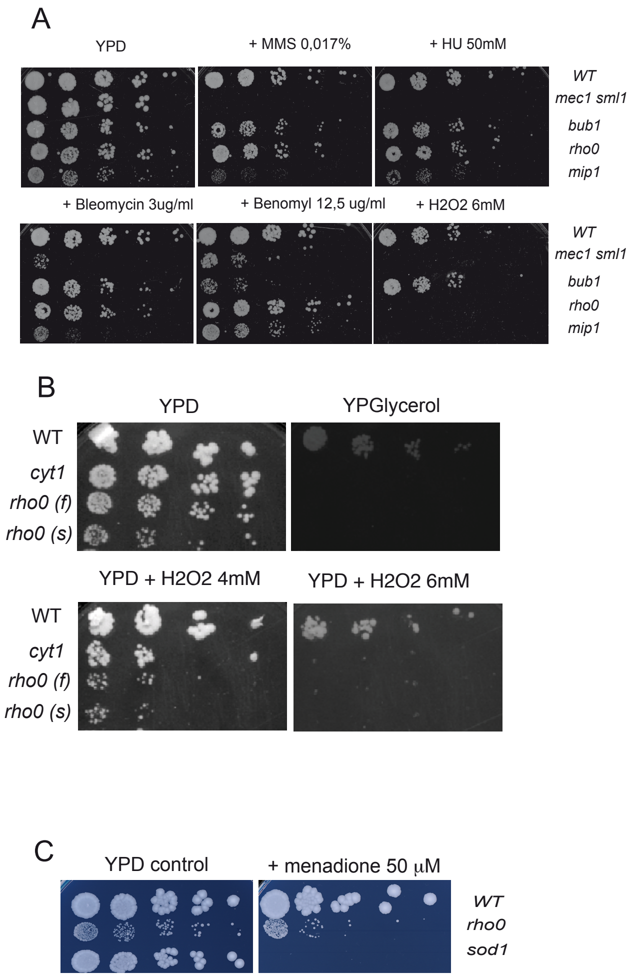

Figure S5 *rho0* cells show hypersensitivity to oxidative stress by H2O2 but not to tested cell cycle or DNA damage drugs. Drop test by serial dilution (5x) of cell suspensions (A) with indicated drugs, added to YEPD plates and insubated for 2 days at 30°. MMS (methymehanesulfonate), HU (hydroxyurea). WT (L1577), *rho0 (f)* (L1994), mitochondrial DNA polymerase mutant *mip1* (*rho0 (s)*, L 1779). Checkpoint mutant controls: *mec1 sml1* (L1734), *bub1* (L1783) (B) Drop test with WT (L1937), *cyt1 RHO+* (L1799) spontaneous *rho0 (s)* strain (L2232), *rho0 (f)* strain (L2249), on YEPD, YEPD + H2O2 and YEPGlycerol plates (C) *rho0* are not hypersentive to oxidative stress by superoxide generating agent menadione. WT (L1937), spontaneous *rho0 (s)* (L2232) and *sod1 RHO+* (L1995)
