# Supplementary material for: Metabolic and Environmental Conditions Determine Nuclear Genomic Instability in Budding Yeast Lacking Mitochondrial DNA
Source: G3 (Bethesda). 2013 Dec 27;4(3):411–23. doi: 10.1534/g3.113.010108 (PMC3962481; doi:10.1534/g3.113.010108)
Supplement: Supporting Information [file supp_g3.113.010108_FigureS6.pdf]

A

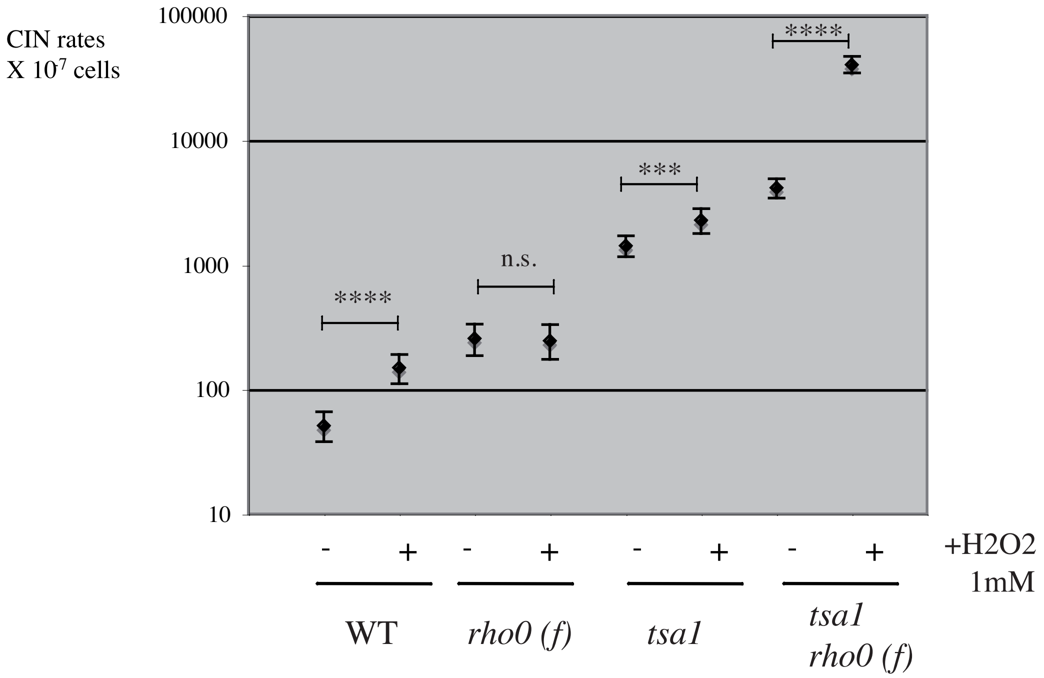


B

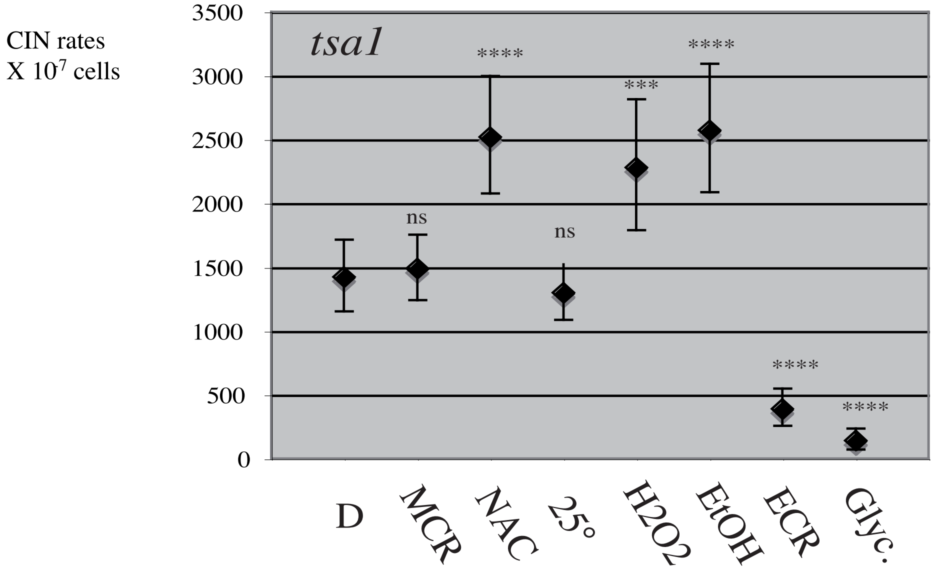


**Figure S6** (A) Highly unstable nuclear genomes in *rho0* cells lacking the peroxiredoxin gene *TSA1*. WT (L1937), *rho0 (f)* (L1994) , *tsa1 RHO+* (L1822) and *tsa1 rho0 (f)* (L1829) were grown on YEPD 2% at 30°, with ot without 1mM H202, and tested by CINAassay. Note the log Y axis. (B) CIN in *tsa1 RHO+* strain (L1822) is high under standard conditions (D=YEPD 30°) and is not reduced by calorie restriction nor by low growth temperature (25°), unlike in *rho0* cells. Ethanol, peroxides and NAC induced a moderate increase of CIN in this strain (less than 2 fold). However, extreme calorie restriction (ECR= 0.05% glucose and YPGlycerol (2%)) leads to a significant stabilization of these cells (4 to 10 fold, $p<0.0001$) compared to standard YEPD 30° conditions.
