# Supplementary material for: Metabolic and Environmental Conditions Determine Nuclear Genomic Instability in Budding Yeast Lacking Mitochondrial DNA
Source: G3 (Bethesda). 2013 Dec 27;4(3):411–23. doi: 10.1534/g3.113.010108 (PMC3962481; doi:10.1534/g3.113.010108)
Supplement: Supporting Information [file supp_g3.113.010108_FigureS7.pdf]

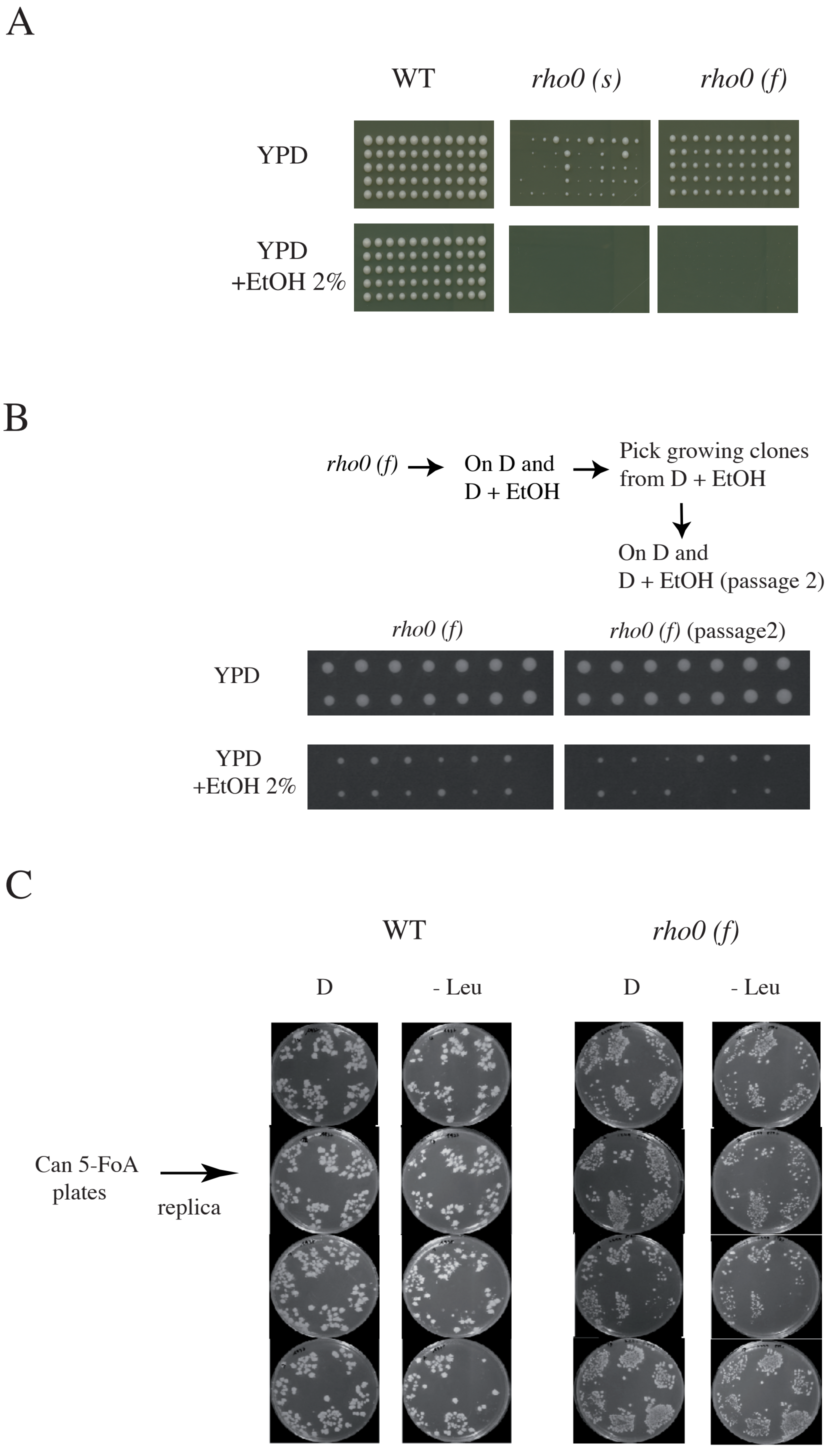


**Figure S7** Ethanol slows down *rho0* colony growth but does not select for suppressors (A) Clonal growth of WT, *rho0 (s)* and *rho0 (f)* CINA strains on YEPD and YEPD + ethanol 2% at 30° for 3 days. (B) One day later, *rho0 (f)* colonies become visible on YEPD+ ethanol and can be tested for CINA (see Figure 6). Individual cells from this plate do not show faster growth when tested again on YEPD + ethanol 2 % (passage 2) , indicating that they are not suppressors. (C) Chromosome breaks and loss in cells grown in the presence of ethanol. WT (L1937) and *rho0 (f)* (L2249) were grown to colonies on YEPD + 2% ethanol and tested by CINA (see Figure 6), then replica plated on - Leu plates to estimate the relative frequency of chromosome breaks (LEU+) versus loss (LEU-).
