# Supplementary material for: Metabolic and Environmental Conditions Determine Nuclear Genomic Instability in Budding Yeast Lacking Mitochondrial DNA
Source: G3 (Bethesda). 2013 Dec 27;4(3):411–23. doi: 10.1534/g3.113.010108 (PMC3962481; doi:10.1534/g3.113.010108)
Supplement: Supporting Information [file supp_g3.113.010108_FigureS8.pdf]

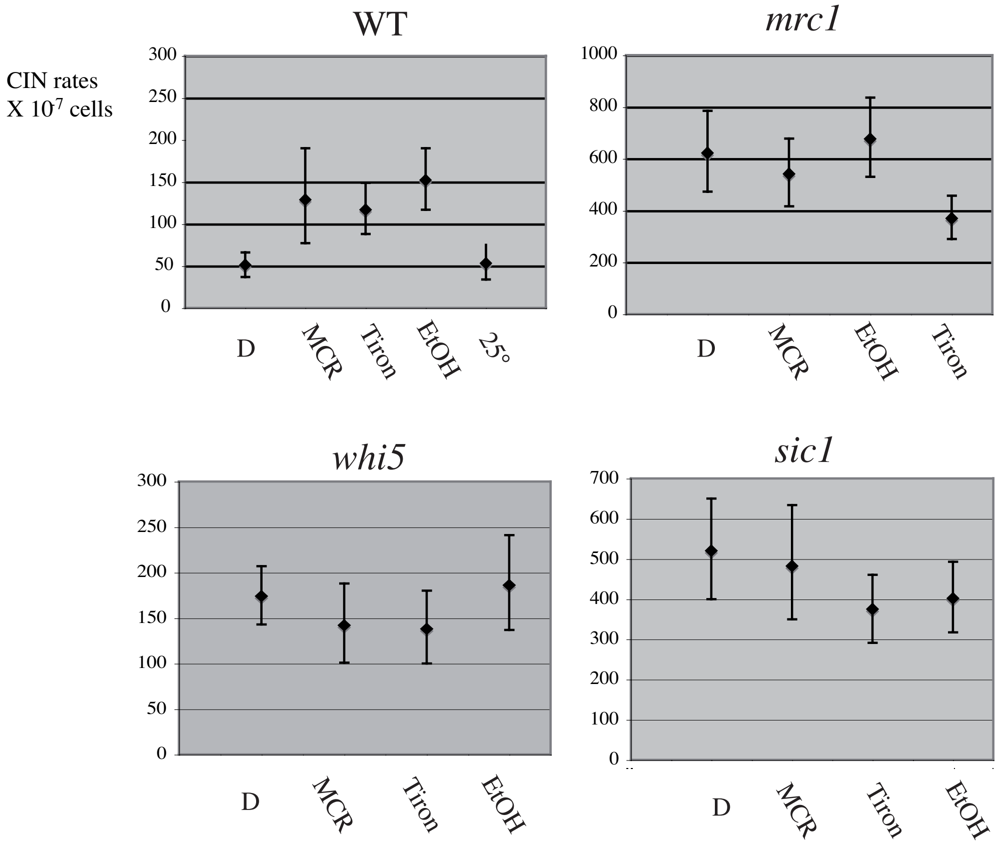

Figure S8 Unlike in *rho0* cells, CIN is fairly constitutive in wildtype and cell cycle mutants grown in various environmental conditions. CINassays were performed on cells deleted for the RB-homolog *WHI5* (*whi5*, L1888), the yeast Claspin homolog *MRC1* (*mrc1*, L1925) and the Cdk/CyclinB inhibitor *SIC1* (*sic1*, L1580). Colonies were grown on plates at 30°. D= YEPD; MCR=moderate calorie restriction (YEP+ 0,5% glucose); Tiron= YEPD + Tiron 1mM ; EtOH= YEPD + 2% ethanol.
