# Supplementary material for: Metabolic and Environmental Conditions Determine Nuclear Genomic Instability in Budding Yeast Lacking Mitochondrial DNA
Source: G3 (Bethesda). 2013 Dec 27;4(3):411–23. doi: 10.1534/g3.113.010108 (PMC3962481; doi:10.1534/g3.113.010108)
Supplement: Supporting Information [file supp_g3.113.010108_FigureS9.pdf]

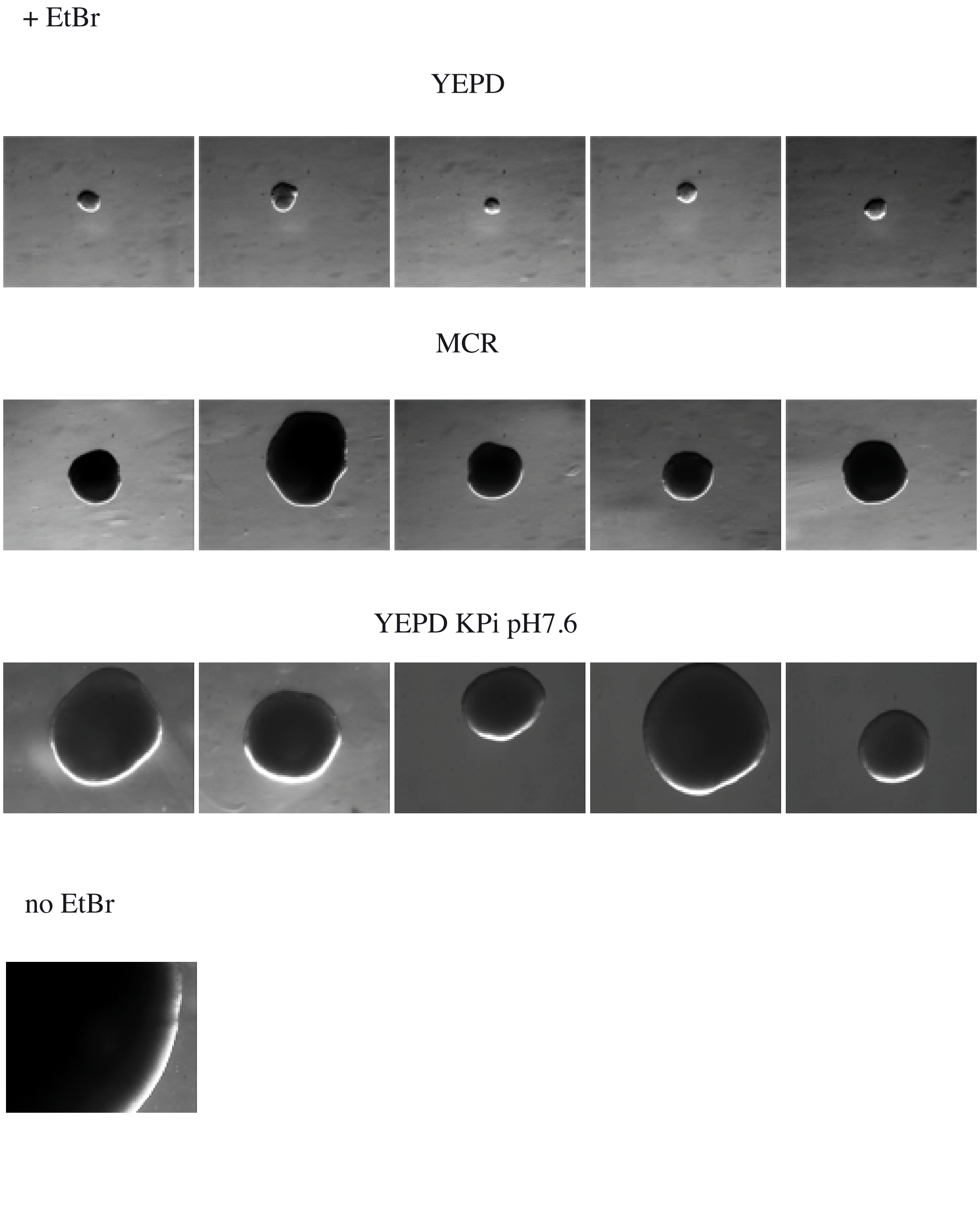

Figure S9 Suppression of the petite-negative *mgr1* mutant by moderate calorie restriction. *mgr1*Δ *RHO+* strain (L 2407) was subjected to ethidium bromide (+EtBr) to induce the loss of mtDNA, and single cells were micromanipulated on unbuffered rich medium (YEPD), under moderate calorie restriction conditions (MCR) or on rich medium buffered at alkaline pH (KPi, pH 7.6), as a positive control. Colony growth at day 4 , 30°. "no EtBr"represents the edge of an untreated *mgr1 RHO+* cell on YEPD. Pictures were taken under light microscope, 10x magnification.
