# Supplementary material for: Metabolic and Environmental Conditions Determine Nuclear Genomic Instability in Budding Yeast Lacking Mitochondrial DNA
Source: G3 (Bethesda). 2013 Dec 27;4(3):411–23. doi: 10.1534/g3.113.010108 (PMC3962481; doi:10.1534/g3.113.010108)
Supplement: Supporting Information [file supp_g3.113.010108_FigureS10.pdf]

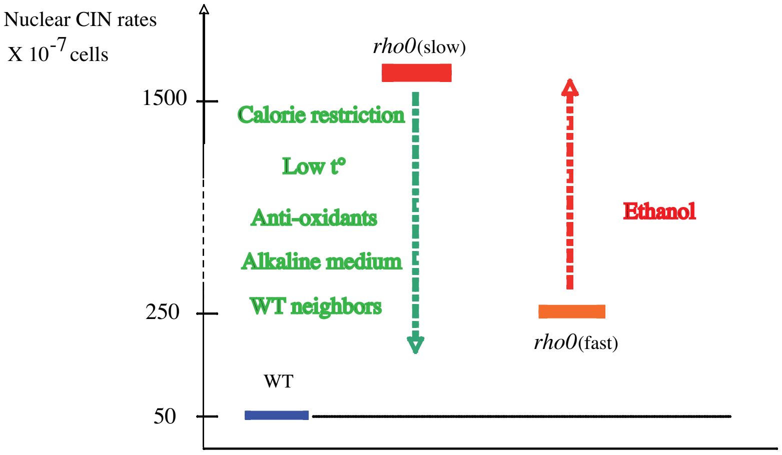

Figure S10 Factors shown to affect instability in *rho0* cells. Bars for WT, slow *rho0 (s)* and fast *rho0 (f)* indicate nuclear CIN in those strains under standard rich conditions (YEPD 30°). Factors in green can stabilize the genome of a *rho0 (s)* close to WT levels. In the presence of ethanol (2%), a moderately unstable *rho0 (f)* strain becomes as unstable as a *rho0 (s)*. Some factors might influence CIN through their effect on mitochondrial membrane potential (affecting ISC-dependent DNA repair), others by their effect on metabolic byproducts (affecting DNA damage?) (see Figure 11).
