# Supplementary material for: Metabolic and Environmental Conditions Determine Nuclear Genomic Instability in Budding Yeast Lacking Mitochondrial DNA
Source: G3 (Bethesda). 2013 Dec 27;4(3):411–23. doi: 10.1534/g3.113.010108 (PMC3962481; doi:10.1534/g3.113.010108)
Supplement: Supporting Information [file supp_g3.113.010108_TableS1.pdf]

Table S1 Strains used in this study

Strain # (L)	Genotype	Origin/Note
1459	*MATa, ura3-52, leu2Δ1, trp1Δ63, his3Δ200, lys2ΔBgl, hom3-10, ade2Δ1, ade8, hxt13::URA3 CAN1*	RDKY3615 (S288C) (CHEN and KOLODNER 1999)
1472	*MATa, ura3-52, leu2Δ1, trp1Δ63, his3Δ200, lys2ΔBgl, hom3-10, ade2Δ1, ade8, hxt13::URA3 rho0*	1459 +ethidium bromide
1520	*MATa, leu2Δ1, trp1Δ63, his3Δ200, lys2ΔBgl, hom3-10, ade2Δ1, ade8, hxt13::URA3 CAN1 ura3-52::LEU2*	1459 with integrated ura3-52::LEU2 (pUL9, F. Cross)
1571	*MATalpha, his3 ::1 leu2::0 met15::0 ura3::0 can1::kanMX 4*	S288C (BY, Euroscarf)
1577	*MATa/alpha ura3-52::LEU2, leu2Δ1, trp1Δ63, his3Δ200, lys2ΔBgl, hom3-10, ade2Δ1, ade8, CAN1, hxt13::URA3/ his3 ::1 leu2::0 met15::0 ura3::0 can1::kanMX 4*	1520 x 1571<br>wild type; CINA strain
1580	*MATa/alpha his3 ::1 leu2::0 met15::0 ura3::0 sic1::kanMX 4 can1::kanMX 4/,ura3-52::LEU2, leu2Δ1, trp1Δ63, his3Δ200, lys2ΔBgl, hom3-10 ade2Δ1, ade8, hxt13::URA3 sic1::HIS3*	*sic1 Δ RHO+*; CINA strain
1607	*MATalpha leu2::0 lys2::0 met15::0 trp1::63 ura3::0 can1*	S288c (BY4727); this study
1690	*MATa/alpha ura3-52::LEU2, leu2Δ1, trp1Δ63, his3Δ200, lys2ΔBgl, hom3-10, ade2Δ1, ade8, CAN1, hxt13::URA3/ his3 ::1 leu2::0 met15::0 ura3::0 can1::kanMX 4 rho0*	*rho0* (s); CINA strain<br>1577 +ethidium bromide; Slow growing
1779	*MATa/alpha his3 ::1 leu2::0 met15::0 ura3::0 trp1 mip1::kanMX4 hxt13::URA3 CAN1 / his3 ::1 leu2::0 ura3::0 can1::kanMX 4 mip1::kanMX4 rho0*	*mip1 Δ rho0 (s)*; CINA strain
1783	*MATa his3 ::1 leu2::0 met15::0 ura3::0 bub1::kanMX4*	*bub1 Δ* ;S288C (BY, Euroscarf)
1799	*MATa/alpha, cyt1::KanMX ura3-52::LEU2 hxt13::URA3 CAN1 (S288c) omnc/ his3 ::1 leu2::0 met15::0 ura3::0 can1 cyt1::kanMX4*	*cyt1 Δ RHO+*; CINA strain
1801	*MATa/alpha his3 ::1 leu2::0 met15::0 ura3::0 can1 clb5::kanMX4/ ura3-52::LEU2 hxt13::URA3 CAN1 clb5::kanMX4*	*clb5 Δ*; CINA strain
1822	*MATa/alpha his3 ::1 leu2::0 met15::0 ura3::0 tsa1::kanMX 4 ura3-52::LEU2 hxt13::URA3 CAN1/ his3 ::1 leu2::0 met15::0 ura3::0 tsa1::kanMX 4 can1*	*tsa1 Δ*; CINA strain
1829	*MATa/alpha his3 ::1 leu2::0 met15::0 ura3::0 tsa1::kanMX 4 ura3-52::LEU2 hxt13::URA3 CAN1/ his3 ::1 leu2::0 met15::0 ura3::0 tsa1::kanMX 4 can1 rho0*	*tsa1 Δ rho0*; CINA strain
1847	*MATalpha ura3-52::LEU2 hxt13::URA3 CAN1 his3 TRP+*	S288C; 1577 spo.
1888	*MATa/alpha his3 ::1 leu2::0 met15::0 ura3::0 whi5::kanMX 4 ura3-52::LEU2 hxt13::URA3 CAN1/whi5::kanMX 4 can1*	*whi5 Δ RHO+;* CINA strain
1925	*MATa/alpha, ura3-52::LEU2 hxt13::URA3 CAN1 his3 mrc1::kanMX4 TRP+ /leu2::0 lys2::0 met15::0 trp1::63 ura3::0 can1 mrc1::kanMX4*	*mrc1 Δ RHO+;* CINA strain

1937	*MATa/alpha ura3-52::LEU2, leu2Δ1, trp1Δ63, his3Δ200, lys2ΔBgl, hom3-10, ade2Δ1, ade8, CAN1, hxt13::URA3/ his3 ::1 leu2::0 met15::0 ura3::0 can1::kanMX 4*	1577; subclone. Wildtype CINA strain
1993	*MATa/alpha ura3-52::LEU2, leu2Δ1, trp1Δ63, his3Δ200, lys2ΔBgl, hom3-10, ade2Δ1, ade8, CAN1, hxt13::URA3/ his3 ::1 leu2::0 met15::0 ura3::0 can1::kanMX 4 rho0 (s)*	Subclone of 1690; slow growing rho0; CINA strain
1994	*MATa/alpha ura3-52::LEU2, leu2Δ1, trp1Δ63, his3Δ200, lys2ΔBgl, hom3-10, ade2Δ1, ade8, CAN1, hxt13::URA3/ his3 ::1 leu2::0 met15::0 ura3::0 can1::kanMX 4 rho0 (f)*	Subclone of 1690; fast growing rho0; CINA strain
2232	*MATa/alpha, ura3-52::LEU2, leu2Δ1, trp1Δ63, his3Δ200, lys2ΔBgl, hom3-10, ade2Δ1, ade8, hxt13::URA3/ his3 ::1 leu2::0 met15::0 ura3::0 can1::kanMX 4 rho0 (s)*	Spontaneous *rho0* (slow) isolated from 1937; CINA strain
2249	*MATa/alpha, ura3-52::LEU2, leu2Δ1, trp1Δ63, his3Δ200, lys2ΔBgl, hom3-10, ade2Δ1, ade8, hxt13::URA3/ his3 ::1 leu2::0 met15::0 ura3::0 can1::kanMX 4 rho0 (f)*	Spontaneous *rho0* (fast) isolated from 2232; CINA strain
2264	*MATa/alpha his3 ::1 leu2::0 met15::0 ura3::0 atp11::KanMx4 ura3-52::LEU2 hxt13::URA3 CAN1*	*atp11Δ*; CINA strain
2267	*MATa/alpha his3 ::1 leu2::0 met15::0 ura3::0 atp10::KanMx4 ura3-52::LEU2 hxt13::URA3 CAN1/atp10::KanMx4 can1*	*atp10Δ*; CINA strain
2288	*MATa/alpha his3 ::1 leu2::0 met15::0 ura3::0 sΔh4::KanMx4 ura3-52::LEU2 hxt13::URA3 CAN1/his3 ::1 leu2::0 met15::0 ura3::0 sdh4::KanMx4 can1*	*sdh4Δ*; CINA strain
2360	*MATa/alpha, ura3-52::LEU2 hxt13::URA3 CAN1 his3 mms19::KanMx4 TRP+ / can1 mms19::KanMx4*	*mms19 Δ*; CINA strain
2372	*MATa/alpha, ura3-52::LEU2 hxt13::URA3 CAN1 his3 mms19::KanMx4 TRP+ / can1 mms19::KanMx4 rho0*	2360 ; *rho0* (fast); CINA strain
2407	*MATa his3 ::1 leu2::0 met15::0 ura3::0 mgr1::kanMX4*	*mgr1 Δ* ; S288C (BY, Euroscarf)
