# Supplementary material for: Metabolic and Environmental Conditions Determine Nuclear Genomic Instability in Budding Yeast Lacking Mitochondrial DNA
Source: G3 (Bethesda). 2013 Dec 27;4(3):411–23. doi: 10.1534/g3.113.010108 (PMC3962481; doi:10.1534/g3.113.010108)
Supplement: Supporting Information [file supp_g3.113.010108_TableS2.pdf]

Table S2 Nuclear genome instability in wildtype and in respiratory mutants under various growth conditions

genotype	Complex affected	CIN D 30°	CIN D 25°	CIN M.C.R. (30°)	CIN D +EtOH (30°)	Strain# L
wildtype	/	5 ± 2	6 ± 1	12 ± 1	13 ± 2	1937
rho0 slow	RC + F0	145 ± 14	8 ± 1	17 ± 2	NV	1993
rho0 fast	RC + F0	43 ± 14	6 ± 1	18 ± 7	129 ± 6	1994
cyt1	RC III	15 ± 11	24 ± 4	13 ± 3	14 ± 1	1799
sdh4	RC II	19 ± 2	ND	ND	ND	2288
atp10	RC V (F0)	12 ± 1	7 ± 1	8 ± 2	14 ± 1	2267
atp11	RC V (F1)	20 ± 4	ND	15 ± 5	20 ± 3	2264

CIN rates (x 10^{-6} viable cells, Lea and Coulson Method of the Median) RC = respiratory chain complex ; ND= Not Determined ; NV= Not Viable. Conditions : YPD (2%) D 30° and 25° ; MCR, moderate calorie restriction (0,5% D) ; D + EtOH (YPD + 2% ethanol) ; FO/F1= F0/F1 subunit of ATPsynthase/-ase (RC V); *rho0* cells are mutant in RC III-IV and in FO of RC V
